# The PdxR-PdxKU locus involved in vitamin B_6_ salvage is important for group A streptococcal resistance to neutrophil killing and survival in human blood

**DOI:** 10.1128/spectrum.01609-24

**Published:** 2024-11-12

**Authors:** Sarah E. Davis, Meaghan T. Hart, Rezia Era D. Braza, Aolani A. Perry, Luis A. Vega, Yoann S. Le Breton, Kevin S. McIver

**Affiliations:** 1 Department of Cell Biology and Molecular Genetics, Maryland Pathogen Research Institute, University of Maryland, College Park, Maryland, USA; University of Wollongong, Wollongong, Australia

**Keywords:** vitamin B_6_, nutrient acquisition, pyridoxal 5' phosphate, virulence, group A streptococcus, regulation

## Abstract

*Streptococcus pyogenes* (Group A *Streptococcus*, GAS) is a Gram-positive bacterium that inflicts both superficial and life-threatening diseases on its human host. Analysis of fitness using a transposon mutant library revealed that genes predicted to be involved in vitamin B_6_ acquisition are associated with fitness in whole human blood. Vitamin B_6_ is essential for all life and is important for many cellular functions. In several streptococcal species, it has been shown that mutants in B_6_ acquisition exhibited reduced virulence phenotypes and were attenuated during infection. In GAS, B_6_ acquisition is believed to be controlled by the *pdxR-pdxKU* locus, where PdxR is a positive regulator of *pdxKU*, which encodes for a B_6_-substrate kinase and permease, respectively. Mutants in the regulator (Δ*pdxR*) and salvage machinery (Δ*pdxKU*) both exhibited modest growth defects when grown in oxygenated conditions with limited vitamin B_6_ precursors. ∆*pdxR* and ∆*pdxKU* mutants also exhibited an impaired ability to survive when challenged with whole human or mouse blood. This defect was characterized by reduced survival in the presence of human neutrophil-like HL60s, primary polymorphonuclear leukocytes, and antimicrobial peptide LL-37. Promoter analysis showed that PdxR is an autoregulator and activated *pdxKU* in the absence of B_6_. Interestingly, ∆*pdxR* and ∆*pdxKU* mutants were not attenuated in mouse models of infection, suggesting a species-specific impact on virulence. Overall, it appears that *pdxR-pdxKU* is associated with GAS vitamin B_6_ metabolism as well as pathogen survival during encounters with the human innate immune system.

**IMPORTANCE:**

Bacterial pathogens such as *Streptococcus pyogenes* (Group A *Streptococcus*, GAS) must be able to obtain needed nutrients in their human host. Vitamin B_6_ or pyridoxal 5’ phosphate is essential for all life and is important for many cellular functions. In other streptococcal pathogens, B_6_ acquisition has been shown to be important for their ability to cause disease. Here, we show that loss of the putative vitamin B_6_ salvage pathway locus *pdxR-pdxKU* affects GAS pathogenesis when encountering innate immune responses from phagocytic neutrophils and antimicrobial peptides within the host. *pdxR-*pdxKU may contribute to oxygen tolerance through B_6_; however, there appear to be other mechanisms for salvaging vitamin B_6_. Overall, *pdxR-pdxKU* is associated with GAS resistance to the human innate immune response and oxygen tolerance and contributes modestly to B_6_ metabolism.

## INTRODUCTION

Active vitamin B_6_, or pyridoxal 5’ phosphate (PLP), is both an essential and versatile enzymatic cofactor that helps catalyze numerous reactions, including those involved in over 100 PLP-dependent enzymatic activities (1). PLP is directly involved in the activity modulation of eukaryotic and prokaryotic transcription factors (2–5). Additionally, vitamin B_6_ is a potent antioxidant that provides protection against oxidative stress (6–10). PLP-dependent enzymes are involved in many processes, such as amino acid biosynthetic pathways as well as deoxy sugar biosynthesis (11–13). Vitamin B_6_ exists in the form of six pyridine compounds, which consist of pyridoxal, pyridoxine, and pyridoxamine, as well as their 5’ phosphate counterparts (14). The only catalytically active form of vitamin B_6_ is PLP, which can be derived from the other 5 B_6_ variants (15). PLP can be obtained either by *de novo* synthesis or by salvaging it from the environment. In the Group A *Streptococcus* (GAS; *Streptococcus pyogenes*), PLP is predicted to be acquired by a salvage pathway. This pathway consists of a predicted permease (PdxU) that imports pyridoxal, pyridoxine, or pyridoxamine, and a putative substrate-specific kinase (PdxK) that can transfer a 5’ phosphate onto any of these three precursors (16, 17). In GAS, the *pdxKU* operon is hypothesized to be regulated by PdxR, a MocR-family transcriptional activator in many bacteria (4), and its deletion is expected to result in vitamin B_6_ auxotrophy (18). In *Bacillus clausii*, PLP acts as a direct anti-activator of PdxR to modulate the expression of *de novo* synthesis genes *pdxST* in response to intracellular PLP levels (5). In GAS, the *pdxR* and *pdxKU* genes are organized in a similar fashion to the *pdxR* and *pdxST* in *B. clausii*, leading to the hypothesis that the GAS *pdxKU* operon is regulated by PdxR in a PLP-dependent manner.

In some pathogens, PLP contributes to virulence characteristics such as biofilm formation (19, 20). A correlation has been reported between PLP acquisition and virulence for pathogens, such as *Helicobacter pylori, Mycobacterium tuberculosis, Streptococcus pneumoniae,* and *Streptococcus mutans* (3, 21–23). When *de novo* PLP synthesis was lost in the Gram-negative pathogen *H. pylori*, virulence was reduced, and defective flagella led to reduced motility (21). In *M. tuberculosis*, loss of vitamin B_6_ production diminished its ability to colonize host tissue (22). For the Gram-positive *S. pneumoniae*, loss of the PLP biosynthesis genes attenuated infection rates in mice (3). Finally, *S. mutans* depends on the salvage pathway for PLP, and the loss of the PdxR activator of *pdxKU* impacted acid stress resistance and biofilm formation (23). Thus, the availability of vitamin B_6_ directly influences the virulence capability of bacterial pathogens.

GAS is a major Gram-positive pathogen able to cause both benign and life-threatening diseases in humans, including bacteremia, streptococcal toxic shock syndrome, necrotizing fasciitis, pharyngitis (Strep-throat), scarlet fever, and rheumatic fever (24–27). GAS has adapted to the human host, resulting in a relatively small genome size (ca. 1.8 Mbp), and is an auxotroph for several important metabolic nutrients that must be obtained from host tissues during infection (28). Like *S. mutans*, the GAS genome appears to lack a biosynthetic locus for vitamin B_6_ biosynthesis and may rely on the PdxKU salvage pathway to obtain precursors from the host environment. Despite the importance of vitamin B_6_ for the growth and virulence of other important pathogens (3, 19–23), this putative PLP salvage pathway has not been previously investigated in GAS. In a transposon-site hybridization (TraSH) screen for GAS fitness and survival in non-immune whole human blood, we identified the PLP precursor kinase gene *pdxK* (29). Therefore, we sought to investigate the role that the *pdxR-pdxKU* locus played in the fitness and virulence of *S. pyogenes*. In this work, we have used defined deletions in *pdxR* and *pdxKU* to validate the importance of PdxR and PdxKU for survival in whole blood and have demonstrated its importance for survival in neutrophils and resisting the cathelicidin antimicrobial peptide LL-37. Additionally, mutants in *pdxR* and *pdxKU* both had modest growth defects in a chemically defined media with limited PLP precursors in oxygenated conditions. We found that PdxR positively regulates the *pdxKU* operon and negatively regulates its own expression. Interestingly, loss of the PdxR-PdxKU pathway did not attenuate GAS during murine bloodstream infection, suggesting a species-specific impact on virulence.

## MATERIALS AND METHODS

### Bacterial strains and media

The GAS strain 5448 is a clinical isolate representative of the globally disseminated M1T1 serotype (30) and was used as the parental wild-type strain for all mutants unless otherwise indicated. For biofilm assays, GAS strain GA19681 (M6) (31) was used as the parental wild-type strain, and mutants were generated in this background. GAS was routinely cultured on trypticase soy agar (TSA) plates with 5% sheep’s blood (VWR). Liquid cultures were grown in Todd-Hewitt medium with 0.2% yeast extract (THY), chemically defined medium (CDM) lacking carbohydrates (CDM-1, Alpha Biosciences) supplemented with 0.5% glucose or custom CDM lacking pyridoxal, pyridoxamine, and carbohydrates (CDM-3, Alpha Biosciences) supplemented with 0.5% glucose. All cultures were grown at 37°C + 5% CO_2_ unless otherwise indicated. Where indicated, CDM-3 was supplemented with pyridoxal and pyridoxamine to 120 pg/mL or 2 µg/mL. CDM-3 plates were made by combining equal parts autoclaved 3% agar cooled to 60°C and 2× CDM-3 + 1% glucose warmed to 60°C (final concentrations: 1.5% agar, 1X CDM-3, 0.5% glucose), and pyridoxal and pyridoxamine were added as indicated. For biofilm assays, brain heart infusions (BHI) media (BD, cat# 237500) were used to propagate GAS. The *Escherichia coli* strain DH5α was used for plasmid generation and maintenance and was regularly cultured in Luria-Bertani (LB) broth (VWR) at 37°C unless otherwise indicated. Antibiotics were used at the following concentrations: spectinomycin (Sp) at 100 µg/mL for both *E. coli* and GAS, and kanamycin (Km) at 50 µg/mL for *E. coli* and 300 µg/mL for GAS. The growth of GAS was assessed by absorbance using a Klett-Summerson colorimeter (Klett units) or a spectrophotometer (OD_600_). Growth of GAS was assessed through CFU enumeration by sampling cultures at the indicated time points, making serial dilutions in saline, and drop-plating 10 µL of dilutions onto THY agar.

### Molecular genetics

All oligonucleotide primers used in this study were synthesized by Integrated DNA Technologies and are listed in Table 1. Plasmids were isolated with either the Wizard Plus SV Miniprep kit (Promega, cat#A1460) or the QIAGEN Plasmid Purification Midi Kit (QIAGEN, cat#12143). Genomic DNA (gDNA) was extracted from GAS using the Master-Pure complete DNA purification kit for Gram-positive bacteria (Epicentre, cat#MGP04100). Restriction enzymes, Antarctic Phosphatase (New England Biolabs [NEB] cat#M0289), and T4 DNA ligase (NEB, cat#M0202) were used according to the manufacturer’s instructions. PCR was performed using either Taq DNA polymerase (NEB, cat#M0273) or High-Fidelity AccuPrime Pfx DNA polymerase (ThermoFisher, cat#12344032) with *ca*. 1 µg of DNA template and 10 pmol of the appropriate primers. When necessary, PCR products were purified using the Wizard SV PCR Clean-Up System (Promega, cat#A9282). Transformations were performed with a Gene Pulser Xcell Electroporator (Bio-Rad) as recommended by the manufacturer, using prepared electrocompetent cells of *E. coli* or GAS. Sanger DNA sequencing was performed by Azenta Life Sciences (formerly Genewiz, Inc.).

**TABLE 1:** Primers used in study

Name	Sequence	Reference
*∆pdxR, ∆pdxKU mutants*		
Ax_spy_0924.1	cccggatccctagaatttgtgttagtggtagc	This study
Ax_spy_0924.2	ggtgatattctcattttagccataaattgtccccatacagttaagtg	This study
Ax_spy_0924.3	tattttactggatgaattgttttagcttttcttcgtaccattaatataaa	This study
Ax_spy_0924.4	cccggatccacttaacacatgtcactaaaaaag	This study
Ax_Spy_0923.1	cccggatccatagcttccttttccttagtgc	This study
Ax_Spy_0923.2	ggtgatattctcattttagccatagctattacctcttttctataataac	This study
Ax_Spy_0922.3	tattttactggatgaattgttttagtacaagtaccttatcgataaaaaagc	This study
Ax_Spy_0922.4	cccggatcctccattccaaaatccataacacc	This study
KmF	atggctaaaatgagaatatcacc	This study
KmR	ctaaaacaattcatccagtaaaata	This study
AX0922V2	tgttgacgttcataggctgc	This study
AX0923V1	agtgtcataataatgaattggtgg	This study
AX0924V1	tgagattcataagcaacacc	This study
AX0924V2	ttggattgagatattctcaatcc	This study
*pdxKU rescue*		
Spy_0923 v.1	agtgtcataataatgaattggtgg	This study
Spy_0922 v.4	tccattccaaaatccataacacc	This study
KmV.1	tagcaggagacattccttcc	(29)
KmV.2	tggggatcaagcctgattgg	(29)
*∆pdxR complement*		
pdxR_complement_pstI-F	aactgcagagctattacctcttttctataataa	This study
pdxR_complement_BamHI-R	cgggatccaattatgtttttttaatttcaaaacc	This study
*qPCR primers*		
QPCR_pdxR_F	acgattttcacatcctctgg	This study
QPCR_pdxR_R	aaacctggtatcgttgtgct	This study
QPCR_pdxK_F	gcggaatctcttttgagga	This study
QPCR_pdxK_R	gcttcccaaaatggaatgtt	This study
*Luciferase primers*		
PpdxKU-F1-BglI	ggaagatctgttactgtaaacactattttcagct	This study
PpdxKU-R1-XhoI	ccgctcgagagctattacctcttttctataataac	This study
PpdxR-F1-BglII	ggaagatctaggtaaaacaagctcagctttttg	This study
PpdxR-R1-XhoI	ccgctcgagaaattgtccccatacagttaagtg	This study
gDNA contamination primers		
LicT_RevTrans_F	aaaccataatgcggcgatttc	(32)
LicT_RevTrans_R	taaggtggtagttgtaccgc	(32)
RT-PCR Primers		
pdxK_F	aattatgatgaagaagatattagacaggtg	This study
pdxU_R	gctgagtacctatataagctaacagtaag	This study

### Nonpolar deletion of *pdxR* and *pdxKU in* M1T1 GAS

Nonpolar deletion mutants were generated in GAS strain 5448 (M1T1) or GAS strain GA19681 (M6) by replacing the open reading frame (ORF) of the target gene with a promoterless *aphA3* kanamycin-resistance marker using allelic exchange as previously described (33). To generate the ∆*pdxR* allelic exchange construct, flanking DNA fragments were amplified with primers oAX0924.1 and oAX0924.2 (5’ homology) and oAX0924.3 and oAX0924.4 (3’ homology). Fragments were joined in-frame via overlap extension (SOE) PCR to the *aphA3* cassette and ligated into BamHI (NEB, cat #R3136)-digested pCRS (29) to produce plasmid pAX0924. GAS 5448 was transformed with pAX0924 at the replication-permissive temperature of 30°C with selection for the plasmid (Sp, 100 µg/mL) and the mutation (Km, 300 µg/mL), and then grown at the non-replication-permissive temperature of 37°C (Km-resistant) to select for integrants via homologous recombination. Resulting merodiploid strains were passaged at 30°C and plated at 37°C (Km 300) prior to screening for allelic exchange Δ*pdxR* mutants (Sp-sensitive, Km-resistant). Allelic exchange of the *pdxKU* ORF by the *aphA3* cassette was conducted with the same methodology using primers oAX0923.1 and oAX0923.2 (5’ homology) and oAX0922.3 and oAX0922.4 (3’ homology) to produce pAX0922-0923 and generate the GAS mutant strain 5448.Δ*pdxKU*.

A ∆*pdxKU* rescue strain was produced during the allelic exchange process upon reversion of the merodiploid to the antibiotic-sensitive WT 5448 genotype. Strains were verified by PCR, with targeted amplification using verification primers oAX0922V2, oAX0923V1, oAX0924V1, and oAX0924V2 to confirm the absence of the *aphA3* cassette and the presence of the WT *pdxR* gene via sequencing. For plasmid complementation of the ∆*pdxR* GAS mutant, wild-type *pdxR* under the regulation of its native promoter (PpdxR) was amplified from WT GAS 5448 gDNA by PCR using the primers pdxR_complement_pstI_F and pdxR_complement_bamHI_R, and cloned into vector pJRS525 (34) using the restriction enzymes PstI (NEB, cat #R3140) and BamHI to generate the complementation plasmid pPpdxR.

### Growth assays in CDM-3 with PLP precursors

For liquid growth assays, strains were grown statically overnight (14–16 h) at 37°C + 5% CO_2_ in 15 mL conical tubes containing 10 mL THY plus antibiotic if necessary. The overnight cultures were pelleted the following morning by centrifugation at approximately 8,400 × *g* and resuspended in saline. 10 mL of CDM-3 + 0.5% glucose in sealed Klett tubes or 30 mL of CDM-3 + 0.5% glucose in 300 mL nephelo flasks were inoculated to an OD_600_ of approximately 0.075. Pyridoxal and pyridoxamine were added to CDM-3 at 120 pg/mL. Sealed Klett tubes were grown statically at 37°C. Nephelo flasks were not sealed, but the caps were left loose and secured with tape to ensure they remained covered and would not become contaminated. Klett flasks were grown at 37°C, shaking at 75 rpm to facilitate aeration. For plate growth assays, strains were grown overnight in THY, pelleted, and resuspended in saline. Saline suspensions were serially diluted and drop-plated onto CDM-3 plates with the indicated amounts of pyridoxal and pyridoxamine.

### Keratinocyte cultivation and cell-based biofilm assay

Primary human skin keratinocytes were obtained from ATCC (cat# PCS-200–011) and cultivated for 2–5 passages in EpiLife medium (Gibco, cat#MEPI400CA) supplemented with Keratinocyte Growth Kit (ATCC cat# PCS-200–040). For assays, keratinocytes were propagated in 24-well tissue culture (TC)-treated dishes until a confluent monolayer was generated. For preservation, the monolayers were fixed with 10% formaldehyde for 30 min at room temperature and then rinsed 5× with phosphate-buffered saline (PBS). Fixed monolayers were stored in PBS at 4°C.

For the biofilm assay (32), GAS overnight cultures were washed 2× with sterile PBS and resuspended in CDM-1 to a final concentration of 10^5^ CFU/mL. 1 mL of culture was added to a well of confluent fixed keratinocytes in triplicate. Every 12 h, CDM-1 media was carefully replaced. The GAS/keratinocyte plates were then incubated for 48 h at 37°C + 5% CO_2_. At the time of harvesting, media was carefully removed from each well, then washed 2× with 1 mL sterile saline. For sample harvesting and CFU counts, 500 µL of saline was added to each well, and a cell scraper was used to remove the GAS and keratinocytes from the plate. The cell slurry was transferred to a Dounce homogenizer, and each well was additionally washed with 300 µL saline to ensure all material was harvested. After the wash was added to the homogenizer, the tissue was ground for 2 min to help with biofilm disruption. The biofilm slurry was then transferred to a 1.5 mL microcentrifuge tube and thoroughly vortexed for 5 min. Spot dilutions from 10^−1^ to 10^−6^ were performed and CFUs were enumerated.

### Polystyrene surface biofilm assay

Overnight GAS cultures were diluted 1:20 into BHI media in a 24-well polystyrene plate (Costar, cat# 3524). All assays were performed in triplicate wells. After addition of the cells, plates were gently rocked back and forth to evenly disperse the cells. Plates were incubated at 37°C with 5% CO_2_ for 48 h. After incubation, all the culture medium was carefully removed, and any non-adherent cells were gently washed away 2× with 2 mL saline solution. All saline was then removed, and the plates were heated for 30 min at 50°C. 500 µL of 0.5% crystal violet were added to each well, and the plates were incubated for 15 min at room temperature (RT). Unbound crystal violet was then removed by pipet, and 2 mL saline washes with 5 min incubation times were performed a total of 3×. All residual saline was removed, and crystal violet was decolorized with 1 mL of 100% ethanol for 10 min. Spectrophotometer reads were taken at OD_570_.

### Luciferase reporter assays

The promoter activity of *pdxR* and *pdxKU* was assessed using luciferase reporters as previously described (35). Briefly, the plasmid pKSM720 (36) with a multiple cloning site immediately upstream of the firefly luciferase ORF (*luc*) was used as a reporter to measure P_*pdxR*_ and P_*pdxKU*_ promoter activity. 500 basepairs (bp) upstream of *pdxR* was amplified using primers PpdxR-F1-BglII and PpdxR-R1-XhoI via PCR, whereas the upstream region of *pdxKU* was amplified using primers PpdxKU-F1-BglII and PpdxKU-R1-XhoI. Both fragments were digested with the restriction enzymes BglII and XhoI and cloned into pKSM720 also digested with BglII (NEB, cat #R0144) and XhoI (NEB, cat #R0146) to generate pPpdxR-luc and pPpdxKU-luc, respectively. The resulting reporter plasmids were transformed into either WT 5448 or the ∆*pdxR* mutant GAS. Reporters were grown either in CDM-1 (+B6) or CDM-3 (No B6). Overnights grown in THY + spectinomycin were washed, then resuspended 1:20 in 10 mL of either CDM-1 or CDM-3 and grown to late log (Klett 80). In total, 1.5 mL of culture was then centrifuged at 12,000 × *g*, and the pellets were frozen until assayed. The assay was performed using a luciferase kit (Promega, Cat# E1500), and samples were read on a Berthold Centro XS LB 960 luminometer with signal collection lasting 1 sec.

### Reverse transcriptase (RT) quantitative real-time PCR (RT-qPCR) and endpoint RT-PCR

GAS overnight cultures were diluted 1:20 into 10 mL THY in Klett tubes and grown at 37°C + 5% CO_2_ until the late log phase of growth (Klett 80) was reached. Cultures were chilled on ice for 10 min, centrifuged for 10 min at 6,000 × *g*, and the pellets were resuspended either in CDM-1 or CDM-3 and “shocked” for 2 h at 37°C + 5% CO_2_. Cultures were pelleted as above and frozen at −80°C overnight. The next day, RNA was isolated as described previously (37). Frozen cell pellets were resuspended in 700 µL of TRI Reagent (Zymo Research, cat#R2050) and approximately 200 µL of 212–300 µm acid-washed glass beads were added (Sigma, cat#G8772). Cells were disrupted by vortexing for 5 min. RNA was isolated with the Direct-Zol RNA Mini-Prep Plus kit (Zymo Research, cat#R2052) following the manufacturer’s specifications. RNA was then DNase treated with the rigorous two-step treatment of the TURBO DNA-*free* kit (Invitrogen, cat#AM1907) plus murine RNase inhibitor (NEB, cat#M0314) to remove residual genomic DNA. To confirm gDNA removal, PCR using standard Taq buffer and polymerase (New England Biolabs, cat#M0273) with oligos LicT_RevTrans_F and LicT_RevTrans_R was performed on 250 ng of total RNA for 45 cycles. Samples positive for gDNA were retreated with the Turbo DNA *free* kit following the manufacturer’s specifications. For endpoint RT-PCR, cDNA was generated using the LunaScript RT SuperMix Kit (New England Biolabs, cat#E3010G). cDNA was then used for PCR with oligos pdxK_F and pdxU_R (Table 1). Real-time analysis was performed using the Luna one-step RT-qPCR kit (NEB, E3005) on a Roche Lightcycler. Specific qPCR primers for transcript amplification are listed in Table 1.

### Lancefield bactericidal assay

The ability of GAS strains to survive in heparinized human or mouse blood was assessed as previously described (38). Briefly, fresh blood was drawn from human volunteers according to Institutional Review Board (IRB)-approved protocols (see Ethics section). GAS was grown overnight in THY, diluted 1:20 into fresh THY and grown to mid-exponential phase (OD_600_ = 0.1–0.15). Strains were then diluted to 10^4^ CFU/mL, and 50 µL diluent (~500 CFU) was added to 500 µL whole blood (human or mouse). Whole human blood and GAS were co-incubated with rotation for 3 h at 37°C. After incubation, the mixture was serially diluted and drop-plated onto square THY agar plates. Multiplication factor (MF) was calculated by dividing the CFU obtained after growth in blood by the initial CFU inoculum. Data are presented as percent growth in blood using the following formula: % growth = (MF of mutant/average MF of WT × 100). Assays can only be completed if the donor is not immune to serotype M1T1 strains, which is indicated by whether the donor blood significantly or completely kills WT 5448. If donor blood results in significant killing of WT, it is excluded from the analysis to ensure that any survival and growth differences, positive or negative, can be detected between WT and the mutant. Mouse blood was acquired from five 8- to 10-week-old female CD-1 mice (Charles River) that were anesthetized with ketamine and xylazine and terminally bled via the brachial artery according to protocols approved by the Institutional Animal Care and Use Committee (IACUC) (see Ethics section). Mouse blood was immediately heparinized and used in the same bactericidal assay described above.

### Growth in human plasma

GAS survival was assessed by vortexing overnight bacterial cultures grown in THY for 10 min. Vortexed cultures were diluted 1:100, and 15 µL of diluent was added to 1,485 µL RPMI + 10% or 50% pooled, heparinized human plasma (Biochemed Services). Samples were taken at t = 0 and every subsequent hour, serially diluted 10^0^ to 10^−5^ and drop-plated onto square plates. The next day, CFUs were counted, and CFU/mL was calculated.

### HL60 culturing and PMN isolation

The HL60 cell line is a human promyeloblast line isolated from peripheral blood that can be differentiated into neutrophil-like cells and was obtained from ATCC (cat# CCL-240). Cells were routinely cultured in RPMI (Hyclone, cat# SH30027.01) supplemented with 10% fetal bovine serum (FBS) + penicillin and streptomycin. During differentiation, cells were washed and resuspended in RPMI + 1 µM all-trans retinoic acid (ATRA) (Sigma, Cat# R2625) at a final concentration of 4 × 10^5^ cells/mL and incubated for 5 d to allow differentiation. After differentiation, cells were pelleted, the supernatant was discarded, and cells were resuspended in RPMI alone to 1.25 × 10^6^ cells/mL.

To isolate polymorphonuclear lymphocytes (PMNs), whole human blood was acquired from anonymous donors via venous puncture and collected in sodium heparin-treated tubes (Fisher, cat# 02–689-6) according to IRB-approved protocol (see Ethics section). The same day, isolation of neutrophils/PMNs was performed using Polymorphprep (Fisher, cat# NC0863559), following manufacturer instructions. Neutrophils/PMNs were then resuspended in RPMI alone to a final concentration of 1.25 × 10^6^ cells/mL and plated at 800 µL/well into a 12-well TC-treated plate (Corning, cat#25815).

### HL60 or PMN survival assay

These assays were performed as described previously (39) with slight modifications. After differentiation of HL60 cells or isolation of PMNs, cells were resuspended into RPMI to a final concentration of 1.25 × 10^6^ cells/mL and plated 800 µL/well in a 12-well TC-treated plate (Corning, cat#25815) to reach a final concentration of 1 × 10^6^ cells/mL. Overnight cultures of GAS were diluted 1:20 into 10 mL THY and grown to an OD_600_ of 0.4. Cultures were then placed on ice for 10 min and pelleted by centrifugation for 10 min at 6,000 × *g*. Media was removed, and the cells were resuspended in 10 mL saline. The GAS cultures were vortexed for 5 min to break the chains, then further diluted 1:10 in saline. To opsonize, 1:10 dilutions were further diluted 1:10 into 1 mL pre-warmed, pooled human plasma (Biochemed Services) and incubated at 37°C + 5% CO_2_ for 30 min. 200 µL of the opsonized bacteria (ca. 1 × 10^5^ CFU) were added to either HL60 cells or PMNs at an MOI of 0.2 and swirled gently. For no HL60 or PMN controls, GAS was added to an RPMI-only well. Plates were centrifuged for 5 min in a swinging bucket rotor at 500 × *g* to ensure the bacteria and mammalian cells were in close contact, then incubated at 37°C + 5% CO_2_ for 2 h.

After incubation, the GAS was harvested using a two-step method. First, the supernatant was removed and stored on ice. Then, 1 mL of deionized water (diH_2_O) was added to each well and vigorously pipetted to liberate any phagocytosed GAS. Control wells with bacteria only were also vigorously pipetted and stored. diH_2_O samples were spun at 9,000 × *g* for 4 min, the supernatant was removed, and the pellet was combined with the 1 mL of media set aside earlier. These tubes were vortexed for 5 min to ensure the lysed HL60’s or PMNs were fully homogenized. The supernatant and the corresponding diH_2_O water lysed samples were combined, then diluted and drop plated (10^−1^, 10^−2^, 10^−3^) onto square THY agar plates (with appropriate antibiotic, if needed) and incubated overnight at 37°C + 5% CO_2_. The next day, CFUs were counted. CFUs were first used to calculate the portion of bacteria that survived compared with the non-HL60 control wells (survival = CFU_HL-60_/CFU_Media-Only_). Then, this metric was normalized to the average WT survival for each experiment and multiplied by 100 to indicate the percent of strain X that survived compared with the WT average (% survival = [survival_X_./avg-survival_WT_]*100). WT was also analyzed in this way to show the variability of WT survival. This was done to allow for the comparison of experiments across individual days and donors. The data reported are the %survival of each strain.

### LL-37 killing

The LL-37 killing assays were performed as previously described (40). All incubations occurred at 37°C + 5% CO_2_. 10 mL of GAS cultures were grown to stationary phase overnight in 15 mL conical tubes. Overnight cultures were vortexed thoroughly and diluted 1:100 into saline. For each biological replicate, two tubes were set up, one as a control and one as experimental: 2.5 µL of diluted GAS was added to 247.5 µL RPMI +10% THY and incubated for 2 h. After this primary incubation, 4 µM LL-37 was added to the experimental sample, and an equivalent volume of water was added to the control sample and incubated for 4 h at 37°C + 5% CO_2_. Tubes were then vortexed for 5 min to help break GAS chains, and dilutions were drop plated (10^0^-10^−5^) onto square THY agar plates. The next day, CFUs were counted and calculated as a percent of survival, compared with the non-LL-37 control tubes.

### Murine intravenous (*i.v*.) infections

All animal experiments were IACUC-approved (see Ethics section). Overnight cultures of strains were prepared in 10 mL THY with antibiotic, if needed. The next morning, the cultures were diluted 1:20 into 125 mL THY in a sealed nephelo flask, grown at 37°C static to an OD_600_ of 0.55, and then immediately put on ice for 10 min to halt growth. Cultures were then moved to conical tubes, vortexed for 10 min to break the chains, centrifuged for 10 min at 5,000 × *g*, and the resulting cell pellet resuspended in approximately 1.2 mL (ca. 2 × 10^8^ CFU/mL), then diluted to approximately 1 × 10^5^ CFU/mL . Initial cell counts were obtained by use of a Petroff-Hauser slide, and the CFU/mL was determined by serially diluting and drop plating on THY agar plates grown overnight at 37°C + 5% CO_2_. CD-1 outbred mice (Charles River Laboratories, strain 022) were held in a restraint device, and 100 µL of GAS (target ca. 1 × 10^4^ CFU/mouse) were injected into a pre-warmed tail vein using a 1 mL tuberculin needle syringes. Ten mice were used per GAS strain. Mice were monitored twice a day and scored for several morbidity phenotypes, including ruffled fur, lethargy, hunched appearance, and hind leg paralysis. Moribund mice were euthanized by CO_2_ asphyxiation or at the end of the study after day 7. A Mantel-Cox test was used to evaluate the significance of differences between the groups.

### Murine subcutaneous (*s.c*.) infections

All animal experiments were IACUC-approved (see Ethics section) using the protocol described previously (41). Briefly, overnight cultures of 5448 were handled as described above for *i.v*. inoculations to produce the infection inoculum. Outbred CD-1 mice (Charles River Laboratories, strain 022) were anesthetized using an approved ketamine/xylazine protocol, fur was removed from the haunch with depilatory cream, and then mice were injected subcutaneously with 2 × 10^8^ CFU in the haunch using 10 animals per GAS strain. Mice were monitored twice a day and scored for several morbidity phenotypes, including ruffled fur, lethargy, hunched appearance, and hind leg paralysis. Moribund mice were euthanized by CO_2_ asphyxiation or at the end of the study after day 7. Skin lesions were measured for area (cm^2^) at 48 h post-infection using ImageJ software (NIH). For the survival data, a Mantel-Cox test was used to evaluate the significance of differences between groups.

## RESULTS

### *∆pdxR* and *∆pdxKU* mutants have modest growth defects in oxygenated conditions in CDM-3 with limited pyridoxal and pyridoxamine

GAS lacks the genes for biosynthesis of the essential B_6_ vitamer pyridoxyl 5’-phophate (PLP) and is likely dependent on a salvage pathway for PLP precursors (pyridoxal, pyridoxamine, and pyridoxine) from the host. In GAS, the putative genes for this salvage pathway are found in an operon, *pdxKU*, which is adjacent to a gene encoding a putative regulator of the operon, *pdxR*. As *pdxK* was identified in a transposon mutagenesis screen as critical for GAS survival in whole human blood (29), we wanted to further explore the importance of PdxKU and PdxR in pathophysiology. Non-polar deletion mutants of both *pdxKU* (∆*pdxKU*) and *pdxR* (∆*pdxR*) were generated in a clinically relevant M1T1 GAS 5448 (WT) background. As a control, a rescue of the ∆*pdxKU* mutant was generated (*pdxKU^R^*). Due to difficulties in obtaining a rescue strain, the ∆*pdxR* mutant was instead complemented *in trans* using a plasmid harboring the wild-type *pdxR* allele expressed from its native P*_pdxR_* promoter (*pdxR^C^*).

To assess the role of the B_6_ salvage pathway genes for GAS growth, we grew mutants in liquid chemically defined media that lacked any PLP precursors (CDM-3). Surprisingly, WT 5448, ∆*pdxR,* and ∆*pdxKU* all grew in CDM-3 completely lacking any PLP precursors (Fig. S1A). This led to the hypothesis that GAS retains PLP stores for use when precursors are not available. Because Δ*pdxKU* lacks the putative PLP salvage genes and Δ*pdxR* lacks the putative regulator, it was hypothesized that the mutants would have fewer PLP stores and would be starved for PLP before the WT. To test this hypothesis, WT 5448, ∆*pdxR*, and ∆*pdxKU* strains were grown overnight in CDM-3 and passaged regularly until growth ceased. Over a 72-h period, no significant differences were seen between the strains (Fig. S1B). However, at 86 h, all the Δ*pdxR* replicates fell below the limit of detection (LOD), whereas the WT replicates all had viable CFUs (Fig. S1B and C). One of the Δ*pdxKU* replicates fell below the LOD at this time point, and two of the replicates were at the LOD. This suggests that the Δ*pdxKU* and Δ*pdxR* mutants have modest defects in importing PLP precursors that limit their resilience in the absence of precursors compared with WT.

**Fig 1:**
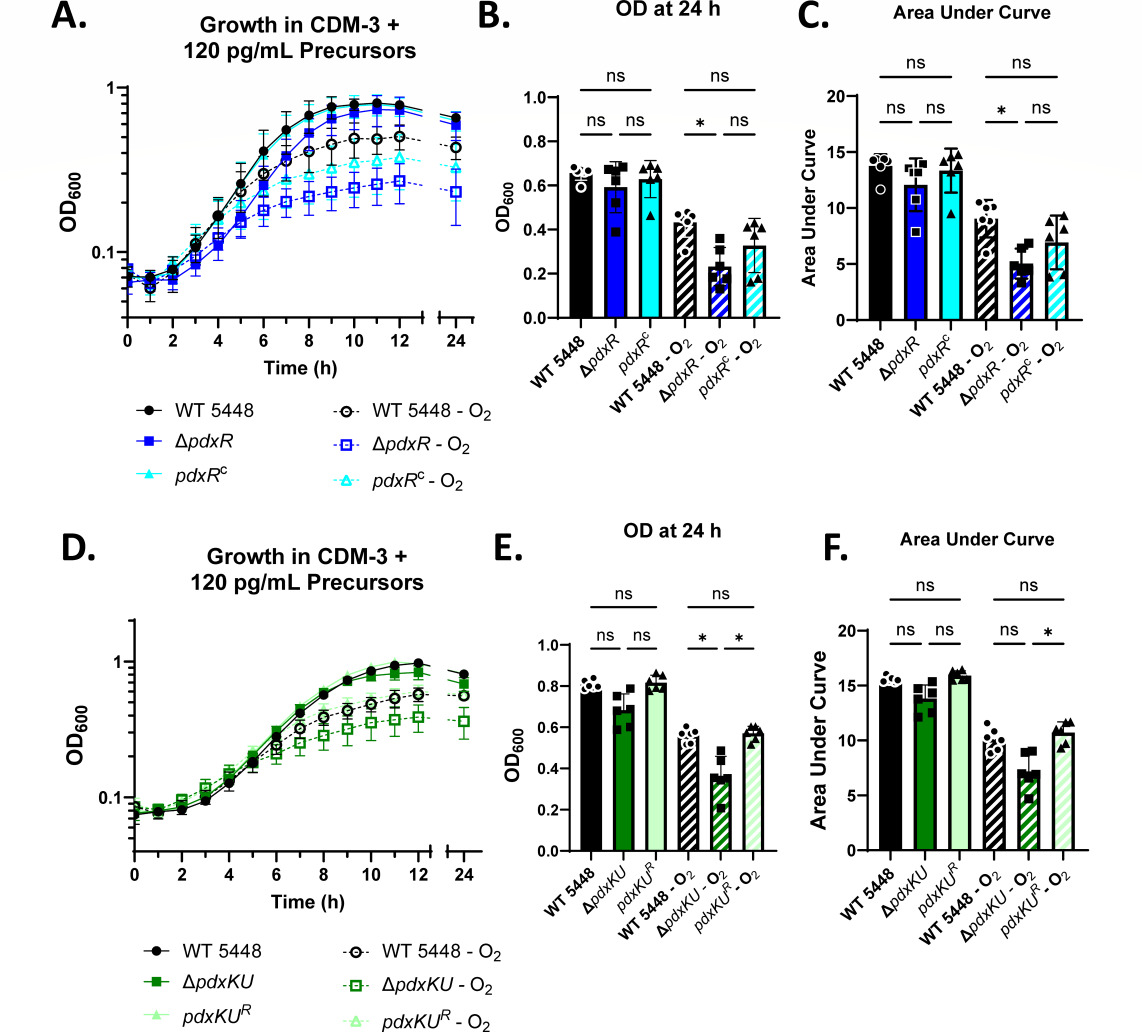
In aerated conditions, Δ*pdxR* and Δ*pdxKU* mutants have a growth defect in CDM-3 plus minimal B_6_ precursors. (A and D) Growth curve of strains grown in CDM-3 + 120 pg/mL pyridoxal and pyridoxamine grown statically in sealed Klett tubes at 37°C (filled symbols, solid lines) and in nephlo flasks with loose lids shaking at 75 rpm (open symbols, dashed lines). (B and E) Bar graph of the OD_600_ of each strain at 24 h. Sealed Klett flasks are indicated by solid bars. Oxygenated flasks are indicated by diagonal striped bars. (C and F) Bar graph of the area under the growth curves. Data represent six replicates analyzed over two independent experiments. Significance was determined by Brown-Forsythe and Welch ANOVA with Dunnett’s T3 test for multiple comparisons (*P < 0.05).

Typically, GAS is grown static in sealed tubes that restrict the amount of oxygen that the bacteria are exposed to (42). Since PLP is a potent antioxidant (6–10), the static and sealed culture conditions may explain why mutant strains were able to grow in CDM-3 lacking PLP precursors. To determine if PLP salvage through PdxR and PdxKU played a role in the growth of GAS in oxygenated conditions, GAS was grown in unsealed nephelo flasks with shaking to facilitate oxygen diffusion. Strains were grown in CDM-3 supplemented with 120 pg/mL of the PLP precursors pyridoxal (PL) and pyridoxamine (PM) (Fig. 1); a concentration chosen because of preliminary drop-dilution plating experiments (Fig. S2). The Δ*pdxR* mutant had a modest growth defect when grown in oxygenated conditions (Fig. 1A through C) as indicated by OD_600_ measurements when the experiment was terminated (Fig. 1B) and area under the curve (AUC) measurements for the full growth curve (Fig. 1C). The Δ*pdxKU* mutant had an even more modest phenotype (Fig. 1D through F). The OD_600_ values at the end of the experiment were significantly lower (Fig. 1E), but the AUC measurements only trended downward and were not statistically significant (Fig. 1F). This growth defect was rescued in *pdxKU*^R^ (Fig. 1E and F), and the *pdxR^C^* complement strain trended back toward WT, although it was not significantly different from that of the mutant (Fig. 1B and C). These data suggest that PdxR and PdxKU play a modest role in tolerating oxygen stress during GAS growth, potentially due to a modest defect in salvaging PLP. Overall, the consistent modest phenotypes in all growth assays suggest that there is another system that contributes to the salvage of PLP.

### Role of PdxR and PdxKU on acid tolerance and biofilm formation

Work on ∆*pdxR* or ∆*pdxST* mutants in *S. mutans* has shown that these genes impact pathogenesis through acid tolerance and biofilm formation (23). In that work, a ∆*pdxR* mutant of *S. mutans* showed reduced survival in acidic media and a significant decrease in biofilm production in comparison to wild-type strains. GAS acidifies its environment over growth, and it must be able to survive pH stress in stationary environments (43). It is also possible that its ability to survive in an acidic environment may be beneficial during infection, for instance, during phagolysosome acidification. Therefore, the ability of GAS to grow in media with different starting pHs was assessed. No differences in the ability of the ∆*pdxR and* ∆*pdxKU* mutants to survive under acidic conditions were observed in comparison to WT 5448 (Fig. S3).

GAS biofilm formation has also been implicated in exacerbating soft tissue infections (44). Since M1T1 5448 does not produce robust biofilms, ∆*pdxR* and ∆*pdxKU* mutants were generated in a high biofilm-producing GAS M6 strain (GA19681), and biofilm formation was assessed. Neither strain exhibited changes in biofilm production on a polystyrene plate (Fig. S4A). It has been shown that GAS is able to adhere specifically to mammalian cells via lipoteichoic acids binding to host albumin, thus promoting biofilm formation (45). To determine if *pdxR* or *pdxKU* plays a role in forming biofilms on mammalian tissue, a biofilm assay on fixed primary human keratinocytes (HEKa) was performed. Again, no significant differences in biofilm formation were observed. (Fig. S4B). Thus, the PdxR-PdxKU locus does not seem to play a major role in survival in acidic media or in biofilm production in GAS.

### PdxR represses *pdxR* and induces *pdxKU* transcription in the absence of pyridoxal and pyridoxamine

**Fig 2:**
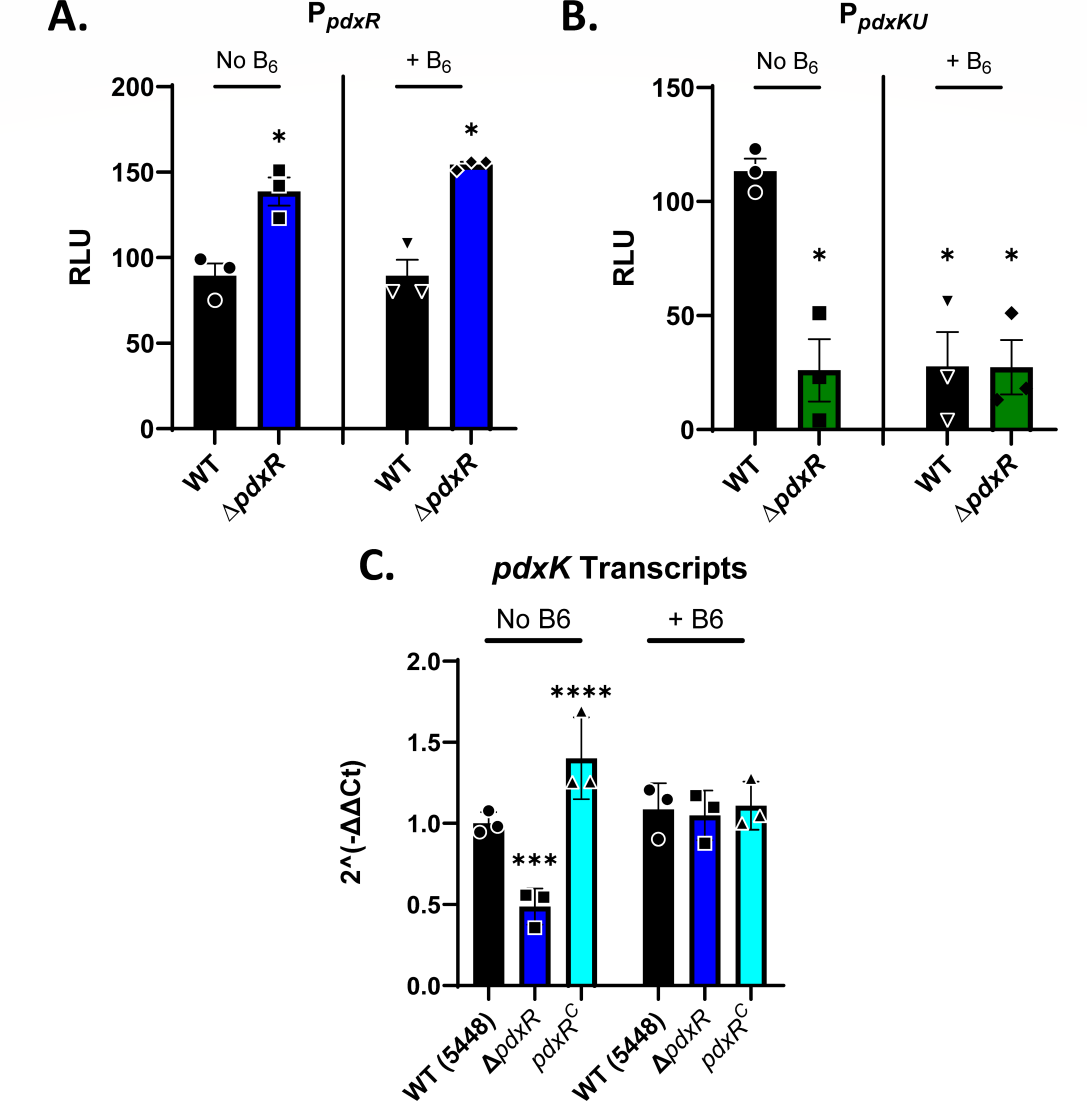
PdxR represses *pdxR* and induces *pdxKU* transcription in the absence of vitamin B_6_ precursors. Firefly luciferase (*luc*) reporter fusions to either (**A**) P*_pdxR_* (blue) or (**B**) P*_pdxKU_* (green) promoters in WT (5448, black) or *∆pdxR* GAS grown in CDM-3 (No B6) or CDM-1 (+B6). Data are expressed as relative light units (RLU). Statistics for A and B were determined by Brown-Forsythe and Welch ANOVA with Dunnett’s T3 test for multiple comparisons. Comparisons shown are to WT with No B_6_ (*P < 0.05) (**C**) qRT-PCR of *pdxK* transcript levels in WT (5448, black), *∆pdxR* mutant (blue), and complemented (light blue) strains grown in THY and shocked in CDM-3 (No B6) or CDM-1 (+B6). Data are reported as 2^(-ΔΔCt) relative WT in no B6 with *gyrA* as an internal control. Statistical significance was tested with two-way ANOVA and Tukey’s multiple comparisons test. Comparisons shown are to WT with No B_6_ (***P < 0.001, ****P < 0.0001).

The PdxR transcription factor has been described as a positive regulator of operons involved in PLP synthesis or salvage such as *pdxKU* (5, 46, 47). and has been shown to have auto-regulatory functions (47). To test the function of PdxR in GAS, we generated transcriptional reporter fusions of the *pdxR* (P*_pdxR_*) and *pdxKU* (P*_pdxK_*) promoters to firefly luciferase (*luc*) in order to measure promoter activity in response to the presence and absence of PLP precursors in the media. The luciferase reporter constructs were transformed into both 5448 (WT) and ∆*pdxR* mutant GAS backgrounds, and promoter activity was measured after incubation in CDM-3 lacking pyridoxal and pyridoxamine (no B6) or in CDM-1, which is formulated with 1 µg/mL of pyridoxal and pyridoxamine (+B6). The *pdxR* promoter (P*_pdxR_*) activity was increased approximately 1.5-fold in the ∆*pdxR* mutant in the presence and absence of pyridoxal and pyridoxamine, suggesting that PdxR modestly represses its own transcription regardless of PLP precursors in the media (Fig. 2A). In contrast, expression at P*_pdxKU_* was down approximately 5-fold in the Δ*pdxR* mutant in the absence of B_6_ precursors (Fig. 2B), suggesting that the PdxR regulator is important for activation of the *pdxKU* promoter (P*_pdxKU_*) when B_6_ is not available in the surrounding environment. When B_6_ precursors were available, there was low luciferase expression from the P*_pdxKU_* promoter.

To confirm these findings by directly measuring transcript levels, the wild-type, the ∆*pdxR* mutant, and the *pdxR^C^* complement strains were grown in THY and then moved to either CDM-1 (+ B6) or CDM-3 (No B6) media for 2 h. Transcriptional start site analysis of the *S. pyogenes* genome predicted that *pdxK* and *pdxU* are transcribed as an operon (48), and RT-PCR confirmed this finding (Fig. S5); therefore, only the transcript levels of *pdxK* were analyzed in RT-qPCR. The ∆*pdxR* mutant exhibited significantly reduced *pdxK* transcripts in the absence of PLP precursors, but not in their presence, compared with WT and the complement strain (Fig. 2C). In contrast with the transcriptional fusion assays, transcripts of *pdxKU* did not decrease when moved to a media with abundant vitamin B_6_ precursors. It is not immediately clear why these two do not agree, but we hypothesize that there may be some kind of post-transcriptional regulation mechanism in the P*_pdxKU_* promoter, which regulates translation. These experiments do suggest, however, that PdxR activates expression of the *pdxKU* B_6_ salvage operon in the absence of PLP precursors.

### Both *pdxR* and *pdxKU* are important for survival and growth of GAS in human blood

**Fig 3:**
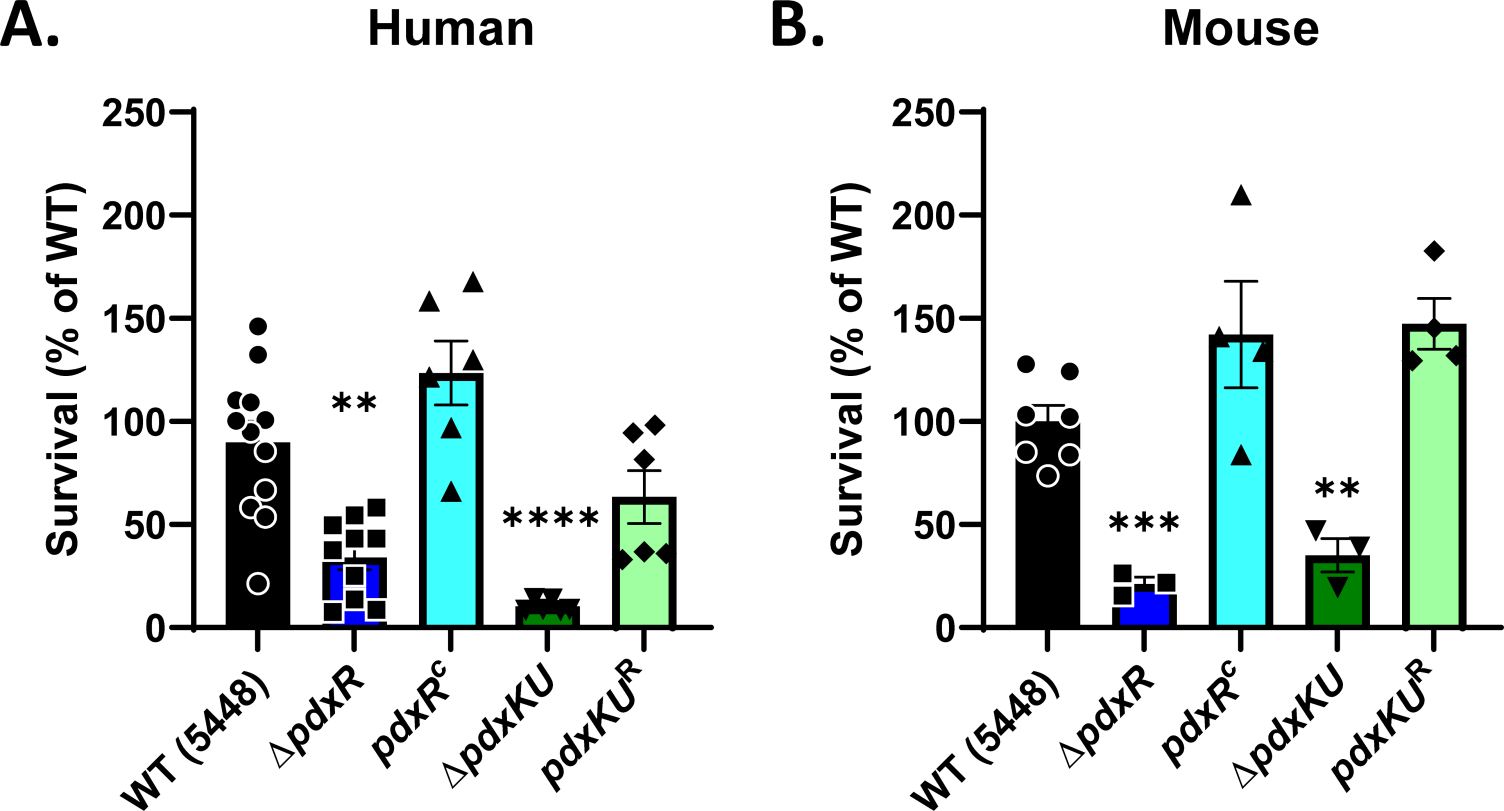
*pdxR* and *pdxKU* are important for survival in both human and mouse whole blood. Lancefield bactericidal assays were conducted on GAS WT (5448, black), the *∆pdxR* (blue) and *∆pdxKU* (green) mutants, and their respective complement (light blue)/rescue (light green) strains. Approximately 500 CFUs of each strain grown to mid-logarithmic phase were co-incubated with (**A**) non-immune human blood or (**B**) non-immune mouse blood from CD-1 outbred mice and then rotated for 3 h at 37°C. Statistics were done by Brown-Forsythe and Welch ANOVA with Dunnett’s T3 test for multiple comparisons. (**P < 0.01, ***P < 0.001, ****P < 0.0001). Error bars indicate the standard error of the mean.

As mentioned previously, *pdxK* was implicated in a transposon screen as a gene important for survival and growth in non-immune human blood (29). To validate this finding and explore the importance of *pdxR* for survival and growth in human blood, the ∆*pdxR* and ∆*pdxKU* mutants and their respective complemented strains were tested using a Lancefield bactericidal assay after 3 h of incubation in heparinized non-immune whole human blood (38). Both the ∆*pdxR* and ∆*pdxKU* mutants had significantly decreased survival as determined by % growth compared with WT and the complemented strains in the assay, confirming that both loci are necessary for fitness in human blood (Fig. 3A). To assess whether there were any species-specific differences between human and mice, we repeated the same assay using whole heparinized mouse blood. Once again, both mutants showed significantly lower % growth compared with WT and the complemented strains (Fig. 3B). Thus, the results were consistent with both *pdxKU* and *pdxR* being required for GAS survival in both human and murine whole blood.

### The *∆pdxR* mutant has a growth defect in the presence of human plasma

**Fig 4:**
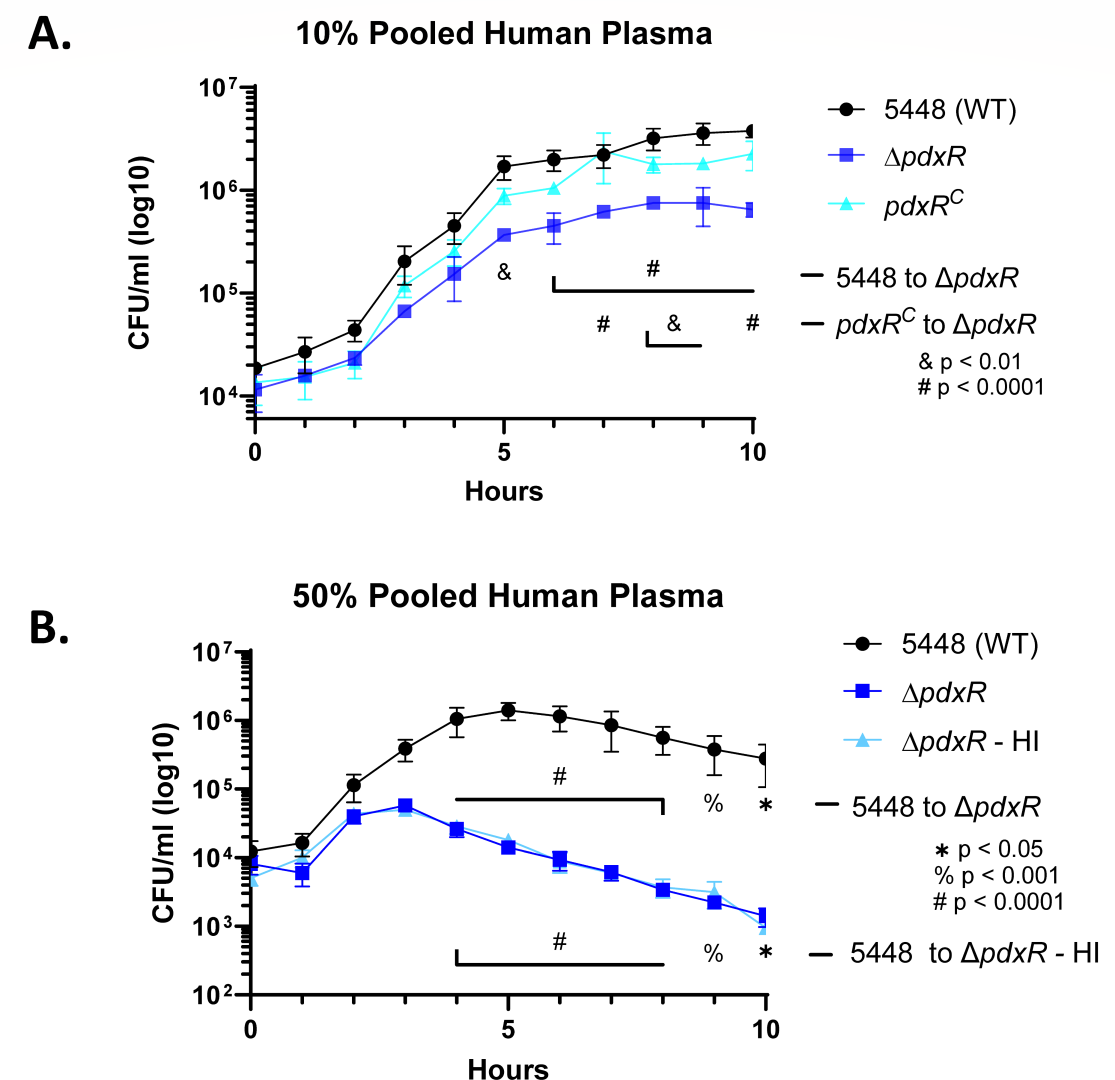
*∆pdxR* has a significant growth defect in the presence of human plasma that is not complement-dependent. (**A**) GAS 5448 (WT, black), ∆*pdxR* mutant (blue), and complemented strain (light blue) were grown in RPMI supplemented with 10% heparinized, pooled human plasma, and growth was determined by plating 10 µL serial dilutions over 10 h. (**B**) GAS 5448 (WT, black) and *∆pdxR* (blue) and complemented strain (light blue) were grown as in (**A**) and in heat-inactivated plasma (HI). Statistics shown are from a Two-Way ANOVA with Tukey’s multiple comparisons test.

Given the *pdxR* and *pdxKU* genes were hypothesized to be involved in the acquisition of an essential nutrient, yet we observed only modest growth defects when grown in oxygenated conditions (Fig. 1), we hypothesized that the defect in blood survival (Fig. 3) was due to an inability to acquire sufficient intracellular vitamin B_6_ specifically in human blood. To evaluate whether the reduced survival in blood was due to the ability to acquire nutrients from this niche, the ∆*pdxR* and ∆*pdxKU* mutants and their respective rescue/complement strains were grown in a physiologically relevant media consisting of tissue culture media (RPMI) + 10% heparinized, pooled human plasma. Interestingly, the *∆pdxR* mutant (Fig. 4A), but not the *∆pdxKU* mutant (Fig. S6) showed a significant defect in growth over time compared with WT and the complemented strain. This growth defect was exacerbated when plasma was increased to 50% (Fig. 4B). When the assay was completed at 50% plasma using heat-inactivated (HI) plasma, the *∆pdxR* mutant was still reduced in survival (Fig. 4B), suggesting that the reduction in survival is not due to complement-mediated killing. The growth defect observed for Δ*pdxR* is not due to minimal PLP availability, as circulating PLP is >5 µg/mL in a healthy individual’s bloodstream (49). The finding that Δ*pdxR* but not Δ*pdxKU* has a growth defect suggests that PdxR regulates genes beyond *pdxKU* that contribute to growth in human plasma. Additionally, Δ*pdxKU* growing similarly to WT in human plasma further suggests that GAS contains an alternative system to acquire PLP.

### *Pdx* mutants are more susceptible to killing by neutrophils and the antimicrobial cathelicidin LL-37

**Fig 5:**
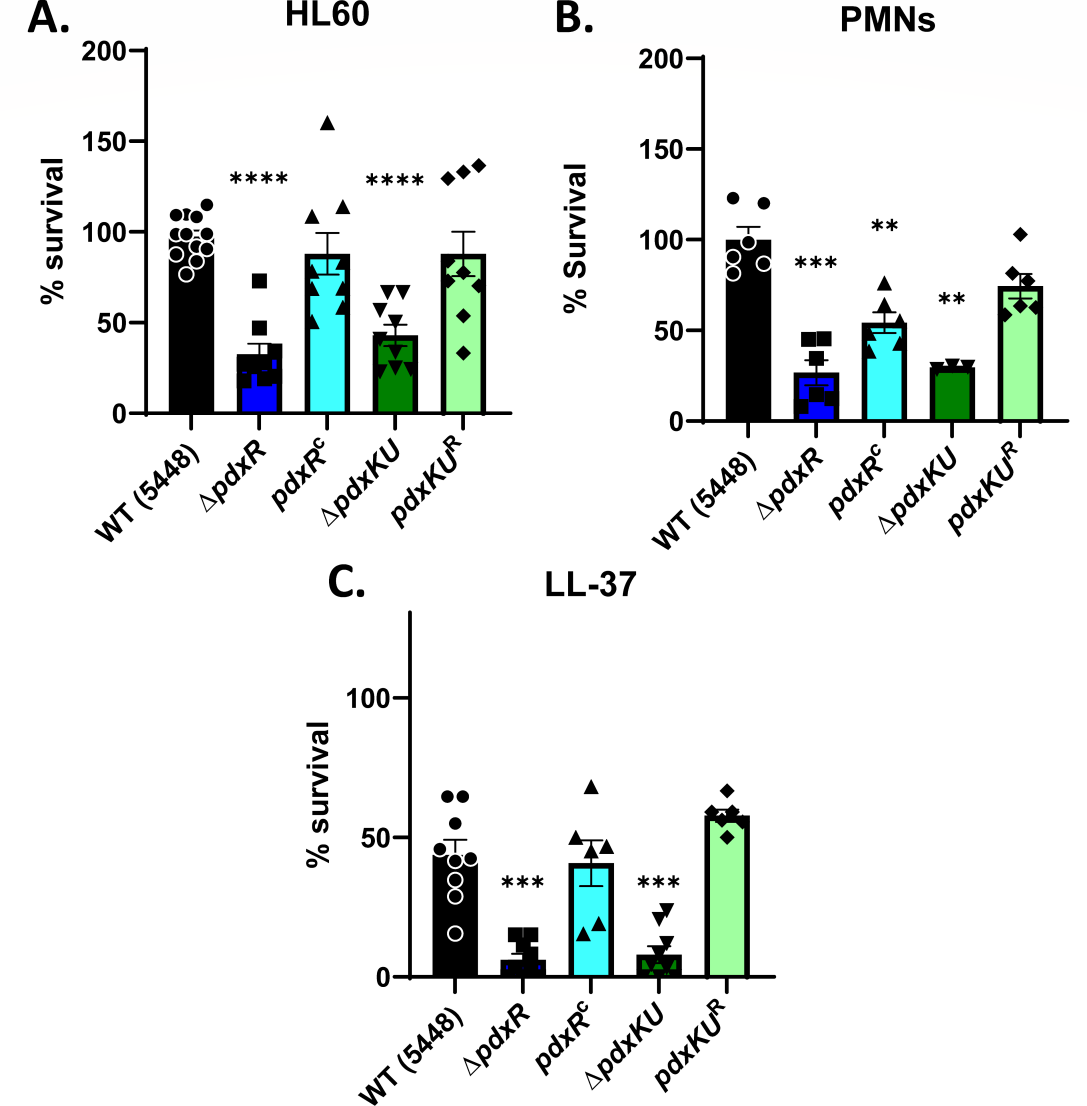
*pdx* mutants are more susceptible to killing by neutrophils and the cathelicidin LL-37. (**A**) differentiated HL60’s or (**B**) human PMNs (1 × 10^6^ each) were incubated with 1 × 10^5^ CFUs of opsonized GAS WT (5448, black), the *∆pdxR* (blue) and *∆pdxKU* (green) mutants, and their respective rescue (light blue)/complement (light green) strains for 2 h. Data are reported as the percent survival of the bacteria compared with the average survival of the WT for each individual experiment, and multiple experiments are reported. See Materials and Methods for a detailed description. (**C**) GAS grown in RPMI + 10% THY were incubated with 4 μm LL37 for 4 h at 37°C, and % survival was determined as in (**A, B**). Data shown represent at least three biological replicates. Groups were compared using a Brown-Forsythe and Welch ANOVA with Dunnett’s T3 test for multiple comparisons (*P < 0.05, **P < 0.01, ***P < 0.001 ****P < 0.0001).

The ∆*pdxR* mutant showed a significant growth defect in pooled human plasma over an extended period, but this was not seen for the *∆pdxKU* mutant (Fig. 4; Fig. S6). Since both mutants exhibited a defect in survival in whole blood, we hypothesized that the *pdx* mutants may have a defect in evading innate immunity. As neutrophils are the most abundant leukocytes in the bloodstream (50) and have been shown to be important for the innate immune response to GAS during infection (51, 52), we looked for differences in survival from neutrophil killing. The HL-60 cell line is derived from blood leukocytes and upon differentiation induced by all-trans retinoic acid (ATRA), they behave like granulocytes (53, 54). To ask whether the ∆*pdxR* or ∆*pdxKU* mutants and their respective rescue/complement strains were susceptible to HL-60 killing, they were grown to mid-logarithmic phase, opsonized with pooled human plasma, and then subjected to HL-60 co-incubation for 2 h. Analysis revealed that the *∆pdxR* and *∆pdxKU* mutants were more susceptible to HL-60 killing, in comparison to WT and their respective rescue/complement strains (Fig. 5A). To explore if this finding translated to differences in killing when encountering primary human neutrophils (PMNs), the assay was repeated with freshly isolated PMNs from human blood. Once again, the ∆*pdxR* and ∆*pdxKU* mutants had a defect in survival, when compared with WT and their complement strains. (Fig. 5B). Thus, both PdxR and PdxKU are important for GAS to survive interactions with human neutrophils.

Antimicrobial peptides are also important innate immune mediators that target and kill bacteria by disrupting their negatively charged membranes (55, 56). The human antimicrobial peptide (AMP) LL-37 (a cathelicidin) is known to lyse GAS by membrane disruption and is also known to trigger pro-inflammatory effects in the host (55, 57–59). LL-37 is constitutively secreted into the blood via innate immune cells and thus is found in human blood and plasma (60). We speculated that LL-37 may contribute to the survival defect of the *∆pdxR* mutant in human plasma (Fig. 4) and in whole human blood (Fig. 3). When strains were grown to early logarithmic phase and then exposed to 4 µM LL-37 for 4 h, both the *∆pdxR* and *∆pdxKU* mutants showed significantly lower survival in comparison to wild-type and their respective rescue/complement strains (Fig. 5C). Although the mechanism involved is unclear, considering Δ*pdxKU* did not exhibit sensitivity to growth in human plasma, Δ*pdxR* and *ΔpdxKU* mutants seem to have increased sensitivity to LL-37 that likely contributes to their overall reduced fitness in blood.

### PdxR and PdxKU are not necessary for bloodstream or soft tissue infections in mice

**Fig 6:**
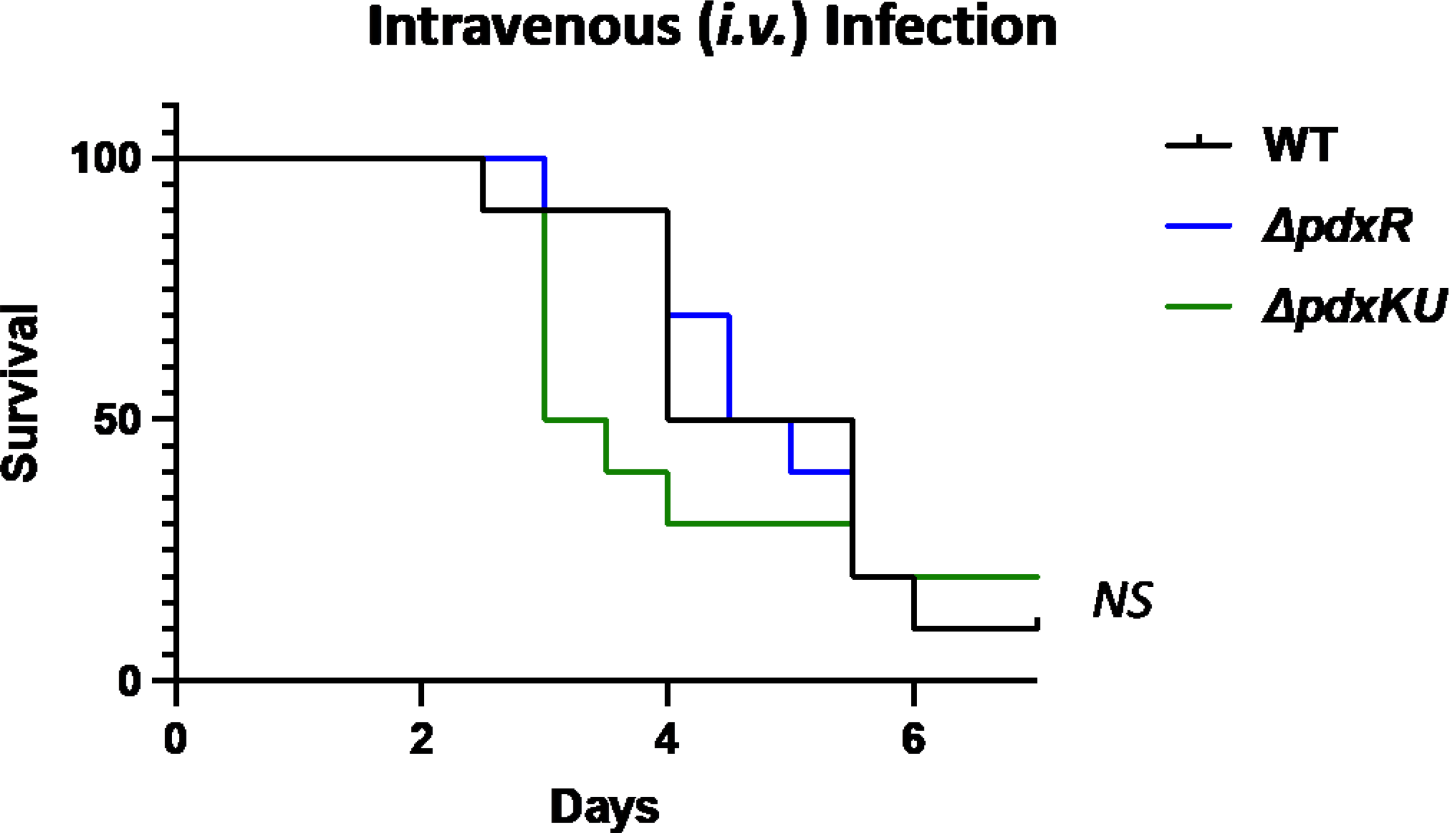
*∆pdxR* and *∆pdxKU* mutants are not attenuated in a mouse model of GAS bloodstream infection. Five × 10^3^ CFU/mouse were injected with either WT (5448, black), ∆*pdxR* (blue), or ∆*pdxKU* (green) via tail vein into CD-1 outbred mice and animals monitored for morbidity over the course of 7 days. A total of 10 mice/strain were used and significance was measured via Kaplan-Meier survival analysis and the log rank test. (NS = non-significant).

∆*pdxR* and ∆*pdxKU* mutants have a greater susceptibility to killing by the innate immune system, and the *∆pdxR* mutant is reduced in the ability to survive in the presence of RPMI + human plasma. To ascertain whether these differences also translate to differences in pathogenesis *in vivo*, an *i.v*. mouse model of bloodstream infection was chosen due to the phenotype of the *pdx* mutants in human and mouse blood, human neutrophils, and with the antimicrobial peptide, LL-37. Outbred CD-1 mice were challenged *i.v*. with 5 × 10^3^ CFUs of either 5448 (WT) or the *∆pdxR* and *∆pdxKU* mutants and monitored for morbidity over a 7-day period. Interestingly, although mice infected with *∆pdxKU* exhibited trends of earlier morbidity, no significant differences in survival were seen overall (Fig. 6). This was surprising as both mutants had significant growth defects in both mouse and human blood as well as decreased survivability when exposed to HL-60s and PMNs. A similar finding was observed when a subcutaneous model of soft tissue infection was performed (Fig. S7A). After infection with 2 × 10^8^ CFU, there was no noticeable reduction in overall survival over the 7-day period or in lesion size and severity 48 h post-infection (Fig. S7B and C). In fact, there was a very modest but statistically significant increase in virulence with the ∆*pdxKU* mutant in soft tissue. Altogether, this suggests that although vitamin B_6_ is important when encountering the human innate immune system, this does not impact virulence in the bloodstream or soft tissue infection of mice.

## DISCUSSION

The purpose of this study was to explore the role of the vitamin B_6_ (PLP) salvage pathway genes (*pdxR, pdxKU*) for GAS pathogenesis during systemic, bloodstream infections. The PdxK pyridoxal kinase gene was identified in a transposon screen as being important for survival in human blood, which led us to hypothesize that the salvage pathway may also be implicated in virulence. PdxR is a known regulator of either the *de novo* biosynthetic (*pdxST*) or salvage (*pdxKU*) genes for PLP acquisition, depending on the bacterium (3, 15, 19, 23, 47). Here, we showed that the *pdxR-pdxKU* gene cluster modestly contributed to GAS growth in oxygenated conditions with limited (120 pg/mL) pyridoxal and pyridoxamine levels (Fig. 1), and PdxR was important for growth in human plasma (Fig. 4). Additionally, the ∆*pdxR* and ∆*pdxKU* mutants were sensitive to survival in whole mouse blood (Fig. 3B). Interestingly, this did not translate to a difference in virulence in a murine model of bloodstream infection (Fig. 6). We also found that both the *∆pdxR* and *∆pdxKU* mutants were sensitive to killing in human blood (Fig. 3A), showed increased phagocytic killing by HL-60 cells and human neutrophils, and were more sensitive to killing by the antimicrobial peptide, LL-37 (Fig. 5). Overall, it appears that *pdxR-pdxKU* plays a modest role in GAS PLP metabolism and oxygen tolerance and a more significant role in pathogen survival during encounters with the human innate immune system.

### *Pdx* mutants have a modest growth defect when B_6_ is limited in oxygenated conditions

As the *pdxR-pdxKU* gene cluster is predicted to be involved in PLP salvage, defined media was used to determine if there were differences in growth for the mutants. Initially, all strains were grown in liquid CDM-3 cultures lacking PLP precursors, and both mutants grew comparable with WT (Fig. S1A). To determine if strains had sufficient PLP stores to facilitate growth over 24 h, strains were passaged continuously in CDM-3 without PLP precursors. At 86 h, Δ*pdxR* and Δ*pdxKU* fell to or below the LOD, whereas all WT replicates were above the LOD (Fig. S1C). These findings suggest that *pdxR* and *pdxKU* contribute very modestly to PLP acquisition.

GAS is considered a facultative or “indifferent” anaerobe and can grow well in the absence or presence of oxygen (61, 62). This aerotolerance quality is predicted to be conferred to GAS through enzymatic NADH oxidase activity (63, 64). PLP is known to be a potent antioxidant and can be protective against oxidative stress (6). For the Gram-negative pathogen *A. pleuropneumoniae,* the loss of PLP synthase enzymes makes it more sensitive to oxidative stress in the form of hydrogen peroxide (65). We speculated that although the Δ*pdxR* and Δ*pdxK* mutants grew like wild type in sealed tubes, they may have growth defects in oxygenated conditions. Both Δ*pdxR* and Δ*pdxKU* had modest growth defects when grown in CDM-3 + 120 pg/mL pyridoxal (PL) and pyridoxamine (PM) in oxygenated conditions (Fig. 1). This suggests that *pdxR-pdxKU* plays a limited role in oxygen tolerance during growth that may be due to its modest role in acquiring PLP precursors. The differences in growth between WT and ∆*pdxR* were not noticeable until pyridoxal and pyridoxamine levels in the media were extraordinarily low, at 120 pg/mL (Fig. S2). In other species such as *S. mutans* (∆*pdxR*) and *S. pneumoniae* (∆*pdxST*), PLP-related mutants had growth defects at much higher concentrations (100 ng/mL levels or greater) of pyridoxal and pyridoxamine (3, 23). The observation that *pdx* mutants have no growth defects until levels of PLP precursors are exceedingly low and oxygen is present suggests that GAS requires much less supplemented PLP.

Our findings that *pdxR-pdxKU* have such modest contributions to PLP acquisition, and oxygen tolerance also strongly suggest that GAS may have other means of PLP acquisition. The GAS genome does not have an annotated or identifiable *pdxST* operon, suggesting that there is no mechanism for *de novo* synthesis of PLP, but it may utilize a redundant salvage pathway. The GAS genome encodes for *thiD* (M5005_Spy_1616), a kinase that has been shown to be able to phosphorylate pyridoxal (66) if PLP precursors can enter the cell through another transporter. This may explain the findings that the contribution of *pdxR-pdxKU* to growth in CDM-3 and oxygenated growth in CDM-3 + 120 pg/mL PL and PM is modest. In future work, this could be further explored by generating a ∆*pdxK*/∆*thiD* conditional double knockout, to see if the loss of both kinases results in a loss of viability. This would suggest that these kinases have redundancy in function and that this phosphorylation activity is essential for PLP acquisition.

### Role of PdxR as a vitamin B_6_-dependent and independent regulator in GAS

RT-qPCR analysis revealed that PdxR positively regulates the *pdxKU* operon, although the impact is modest at approximately 2-fold (Fig. 2C) and that PdxR represses its own transcription in CDM-3 regardless of the levels of pyridoxamine or pyridoxal present (Fig. 2A). PdxR also appears to be an activator of the *pdxKU* operon. This is likely occurring via direct binding of PdxR to an inverted repeat motif present in P*_pdxKU_* (5, 46). In *S. mutans*, *pdxKU* regulation is comparable, with downregulation but not complete loss of *pdx* operon expression in a ∆*pdxR* mutant (23). In contrast, *L. monocytogenes* absolutely requires PdxR for expression of the PLP biosynthetic operon *pdxST* (47). In our findings, GAS appears to act similarly to *S. mutans*, where PdxR only partially activates transcription of *pdxKU* in response to low or missing levels of PLP precursors. The data from the luciferase transcriptional fusion assay (Fig. 2B) support a model where PdxR acts as an activator of *pdxKU* in the absence of PLP. Then, when PLP is present, the expression of *pdxKU* is no longer activated because PLP binds to PdxR and changes its conformation such that it can no longer act as an activator and/or becomes a repressor. However, the RT-qPCR results indicate that there is a similar *pdxKU* transcript when PLP precursors are present as when they are absent (Fig. 2C). This would suggest that there are other factors contributing post-transcriptionally to the regulation of translation of PdxKU when PLP precursors are present. The presence of some kind of PLP-responsive element in the 5′-UTR of P*_pdxKU_* could explain the findings that *pdxKU* transcripts are still abundant, but translation from the promoter is low.

In a physiologically relevant media of human plasma + RPMI, we found that the ∆*pdxR* mutant, but not the ∆*pdxKU* mutant, exhibited a growth defect (Fig. 4; Fig. S5). This suggests that PdxR plays a role in gene regulation beyond *pdxKU*. This regulation could contribute either to nutrient acquisition within the bloodstream or to virulence factors that enable survival in this niche. In future work, it would be interesting to investigate the regulon of PdxR and if this regulon includes nutrient acquisition and virulence operons. Additionally, it would be interesting to determine how PLP availability impacts regulation. PLP is a known cofactor of multiple transcription factors; hence, its availability could lead to global effects on gene expression (1, 4).

### Role of PdxR and PdxKU in immune system survival and virulence

GAS evasion of the immune response is essential for survival in the host, and GAS has developed many mechanisms to evade killing by the many components of immunity (67–69). This study shows that PdxR-PdxKU is important for survival in whole human blood (Fig. 3A), for phagocytic killing (Fig. 5A and B), and for resistance to LL-37 killing (Fig. 5C). These results suggest that the reduced survival of both ∆*pdxR and* ∆*pdxKU* mutants in human blood is due to both the sensitivity to immune cell activity (HL-60s and PMNs) and protein-mediated AMP resistance (LL-37). This study indicated that PdxR-PdxKU does not play a role in acid tolerance or biofilm formation (Fig. S3 and S4); hence, these are not contributing factors to the observed *ex vivo* phenotypes.

The reduced growth of the *∆pdxR* mutant in human plasma was not impacted by heat inactivation (Fig. 4), which suggests that PdxR, but not PdxKU, is important for complement-independent growth in plasma. This may be due to plasma containing cationic antimicrobial peptides (CAMPs), which are notoriously resistant to heat inactivation, although this phenomenon is not LL-37-specific, due to both mutants being susceptible to LL-37 killing (Fig. 5C). Besides LL-37, there are other CAMPs that are predicted to have the ability to target GAS, such as HNP-1 and defensins (70–72), that may influence both *pdx* mutants, which could be explored further.

The reported *ex vivo* phenotypes may be due to the modest regulation of *pdxKU* and the resulting uptake of PLP precursors, but it is also possible that both *pdxR* and *pdxKU* are vital for evading innate immune responses through an undefined mechanism. As mentioned before, PLP is a potent antioxidant that may play a role in quenching interactions with reactive oxygen species (ROS), and the *pdxR* and *pdxKU* mutants both had modest growth defects in oxygenated conditions (Fig. 1). Production of toxic ROS is an important component of the inflammatory response; therefore, the sensitivity of the *pdx* mutants to HL-60 and PMN killing may be ROS-mediated (73). It is curious that there is no difference in survival in mice for the *pdx* mutants (Fig. 6; Fig. S6A), despite there being a proliferation defect in the mouse blood Lancefield (Fig. 3B). This may be because the phenotypes (~50%–75% reduction compared with WT) of the *pdx* mutants in mouse blood, although significant, are not enough to lead to a measurable phenotype *in vivo*. Additionally, the balance of lymphocytes and neutrophils in adult animals is quite different than in humans. Human blood is neutrophil rich (50%–70% neutrophils, 30%–50% lymphocytes), whereas mouse blood has a strong preponderance of lymphocytes (75%–90% lymphocytes, 10%–25% neutrophils) (74). Additionally, the antimicrobial peptide profile is different between mice and humans (75). In humans, α-defensins are the most abundant AMP in neutrophils, whereas α-defensins are not present at all in murine neutrophils (75). Thus, PdxR and PdxKU may play a species-specific role in infections due to the adaptation of GAS to the human host.

### Conclusions

Here, we explored the role of a putative vitamin B_6_ (PLP) salvage pathway (PdxR, PdxKU) for GAS pathogenesis. We found that the *pdxR-pdxKU* gene cluster was important for growth in low levels of PLP precursors in oxygenated conditions, for growth in human and mouse whole blood, and for resistance to killing by neutrophils and the antimicrobial peptide LL-37. PdxR is necessary for the activation of *pdxKU* in the absence of B_6_ precursors and autoregulates its own expression. Interestingly, loss of this putative B_6_ salvage pathway did not lead to attenuation in a murine model of bloodstream infection. Overall, *pdxR-pdxKU* appears to be important for GAS survival during encounters with the human innate immune system.

## SUPPLEMENTAL MATERIAL

Supplemental material spectrum.01609-24-s0001.pdf Fig. S1 to S7.

ASM does not own the copyrights to Supplemental Material that may be linked to, or accessed through, an article. The authors have granted ASM a non-exclusive, world-wide license to publish the Supplemental Material files. Please contact the corresponding author directly for reuse.
