# Supplementary material for: The PdxR-PdxKU locus involved in vitamin B6 salvage is important for group A streptococcal resistance to neutrophil killing and survival in human blood
Source: Microbiol Spectr. 2024 Nov 12;12(12):e01609-24. doi: 10.1128/spectrum.01609-24 (PMC11619246; doi:10.1128/spectrum.01609-24)
Supplement: Supplemental material — Fig. S1 to S7. [file spectrum.01609-24-s0001.pdf]

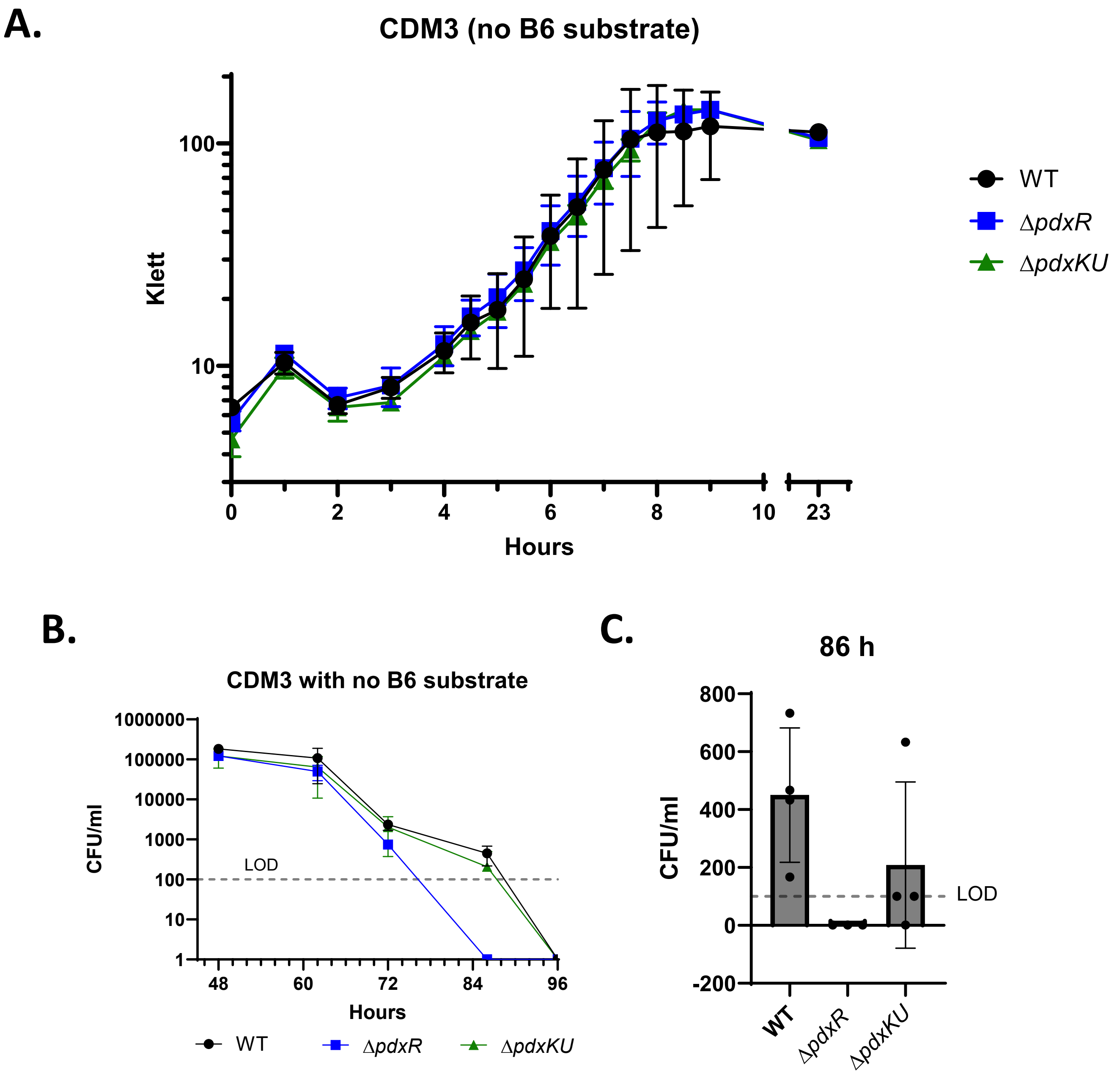

Supplemental Figure S1. Δ*pdxKU* and Δ*pdxR* mutants grow similar to WT in CDM-3 up to 72 hours. (A) Strains indicated were grown in CDM-3 without pyridoxal or pyridoxamine statically in sealed klett tubes at 37°C and absorbance was measured. GAS WT 5448 (black circles), Δ*pdxR* mutant (blue squares) Δ*pdxKU* mutant (green triangles). **(B)** Strains were grown in CDM-3 without pyridoxal and pyridoxamine and growth was quantified by drop plating serial dilutions (10 µl) of cultures at the time points indicated on the X-axis. **(C)** Bar graph of the CFU/mL of each strain at 86 h.

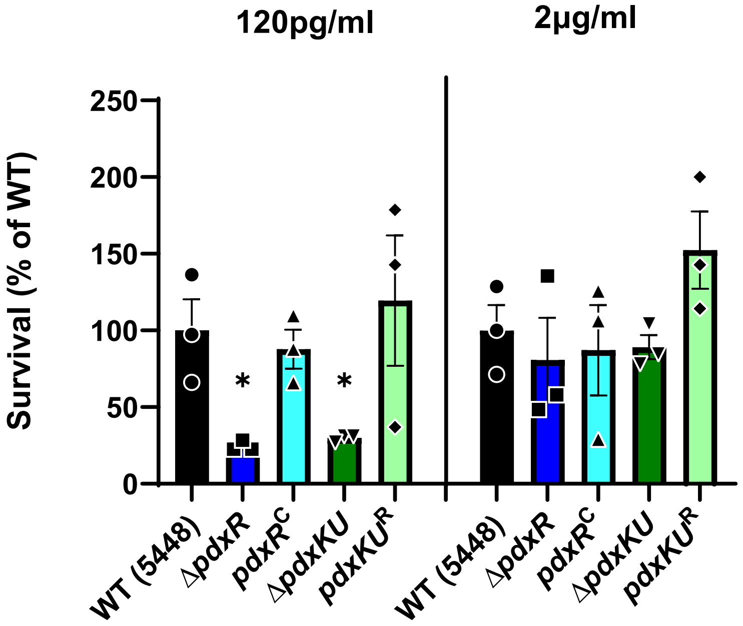

Supplemental Figure S2. Δ*pdxR* and Δ*pdxKU* mutants have a growth defect on solid media containing low B_6 substrate. Overnight cultures of GAS WT (5448, black), the Δ*pdxR* (blue) and Δ*pdxKU* (green) mutants and their respective rescue (light blue)/complement (light green) strains were serially diluted, and spot plated for CFU on CDM-3 agar plates containing minimal levels (120 pg/ml) or high levels (2 ug/ml) of the PLP precursors pyridoxal or pyridoxamine. Data from a single, representative experiment with 3 independent replicates/genotype were expressed as % survival compared to WT. Statistics based on unpaired t-test. (* $p < 0.05$).

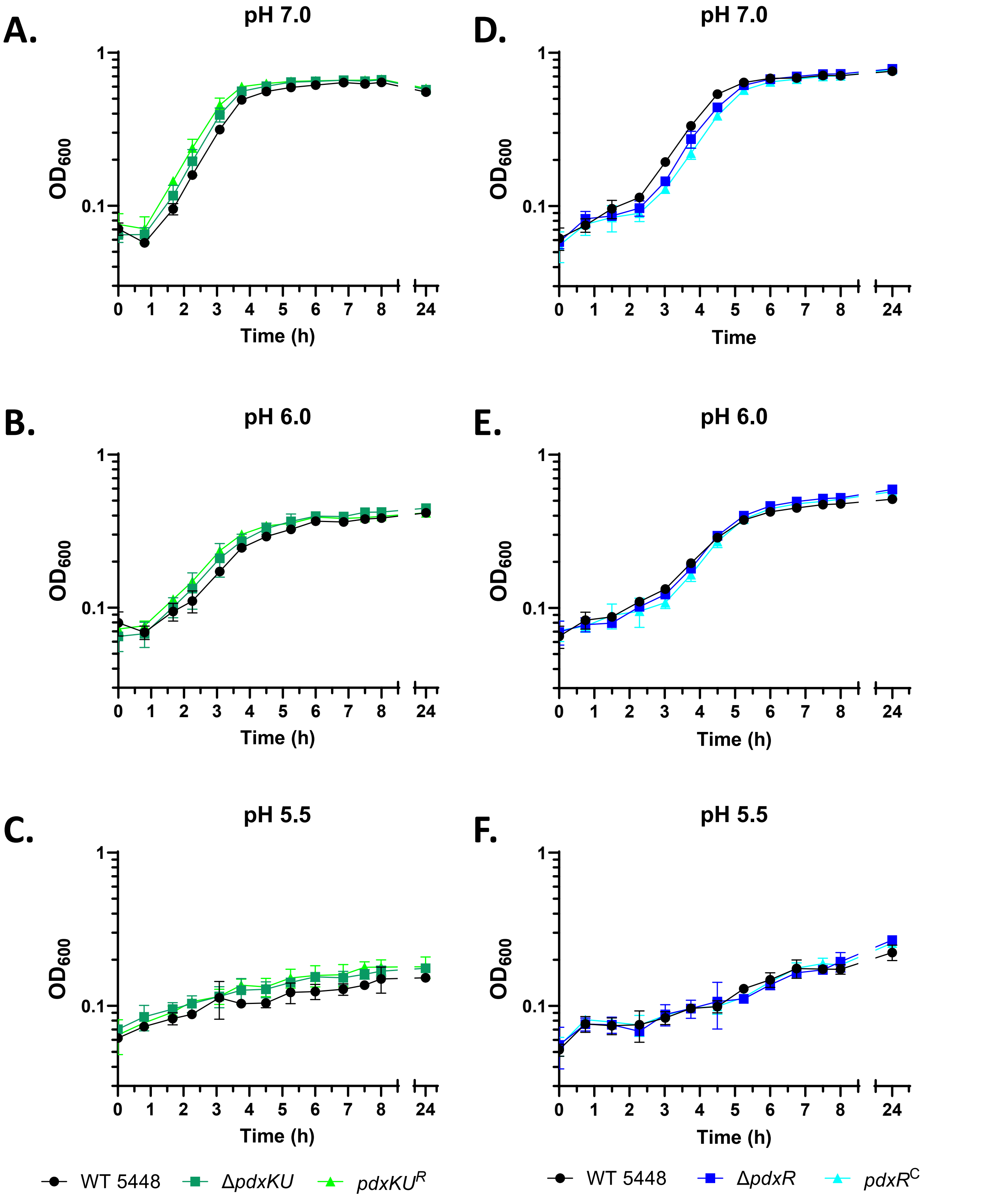

Supplemental Figure S3. No growth phenotype observed for Δ*pdxR* or Δ*pdxKU* mutants in THY at various pHs. (A-C) Growth curves of WT (black circles), Δ*pdxKU* (green squares), and *pdxKU*^{R} (light green triangles) in THY adjusted to the indicated pHs. **(D-F)** WT 5448 (black circles), Δ*pdxR* (blue squares), and *pdxR*^{C} in THY + spectinomycin adjusted to the indicated pHs.

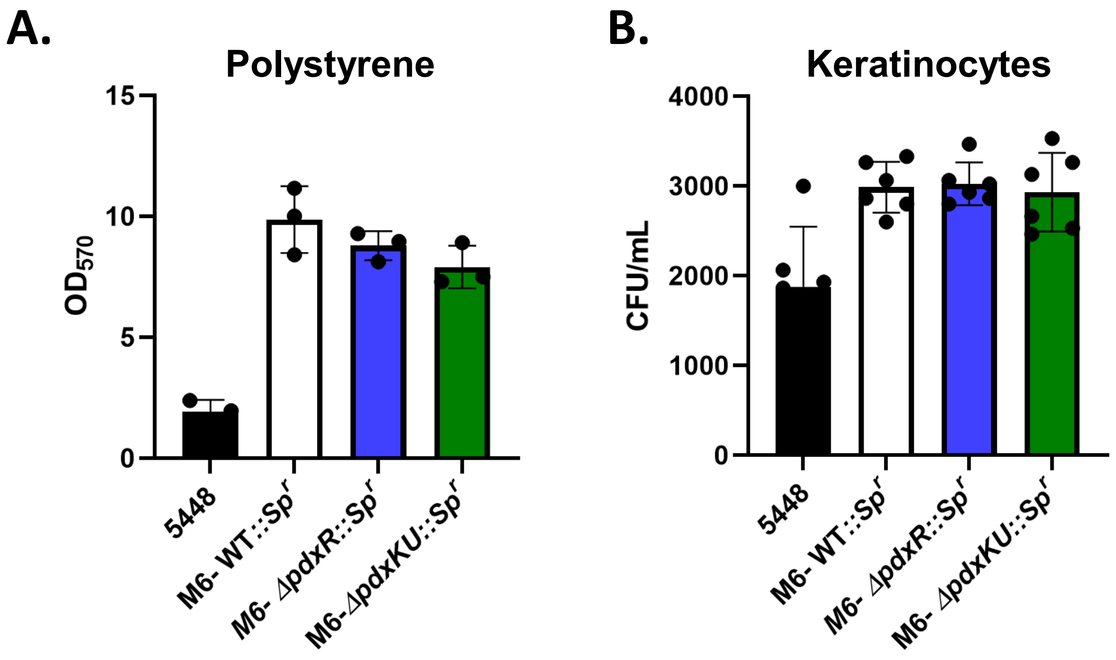

Supplemental Figure S4. No biofilm phenotype for ∆*pdxR* or ∆*pdxKU* mutants in a high biofilm producing background. (A) Crystal violet quantification of biofilm grown in BHI on polystyrene and **(B)** viable biofilm grown on primary human keratinocytes in CDM-1 media. No statistical differences. 5448 was included as a negative control that is a low biofilm-producing strain.

A.

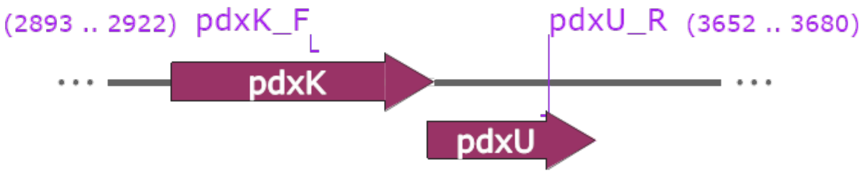

B.

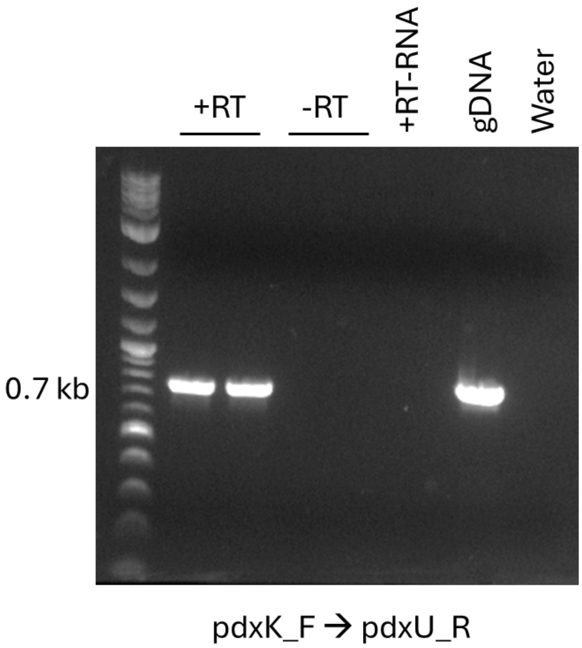

Supplemental Figure S5. *pdxK* and *pdxU* are co-transcribed. A) Schematic of the gene locus with the location of oligos used for RT-PCR. **B)** PCR with oligos pdxK_F and pdxU_R was conducted on cDNA to determine if *pdxK* and *pdxU* were present on a single transcript. +RT indicates samples that contained reverse transcriptase. –RT samples did not contain reverse transcriptase and were included as negative controls for gDNA contamination. +RT-RNA sample was included as a negative control and was the master mix containing reverse transcriptase without any template RNA to control for any contamination within the master mix. The gDNA and water wells were PCR positive and negative controls, respectively. cDNA was generated from RNA collected from WT 5448 grown to mid-exponential phase (OD ~ 0.3) in THY.

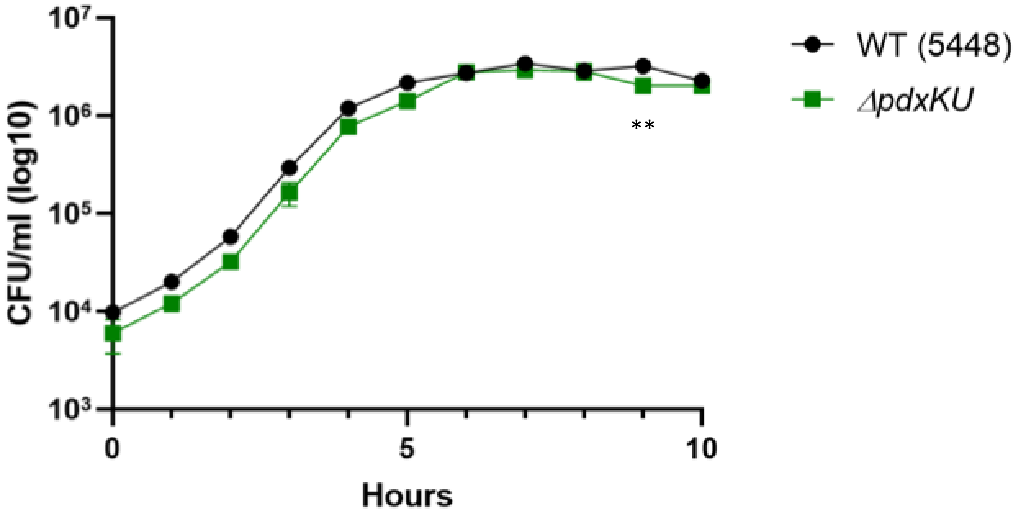

Supplemental Figure S6. Δ*pdxKU* mutant does not have a significant growth defect in the presence of human plasma. GAS WT (5448, black) and Δ*pdxKU* mutant (green) strains were grown in RPMI supplemented with 10% heparinized pooled human plasma, and growth was quantified by drop plating serial dilutions (10 µl) at regular intervals indicated on the X axis. Statistics were performed using a two-way RM ANOVA with Sidak's multiple comparisons test (** $p < 0.01$).

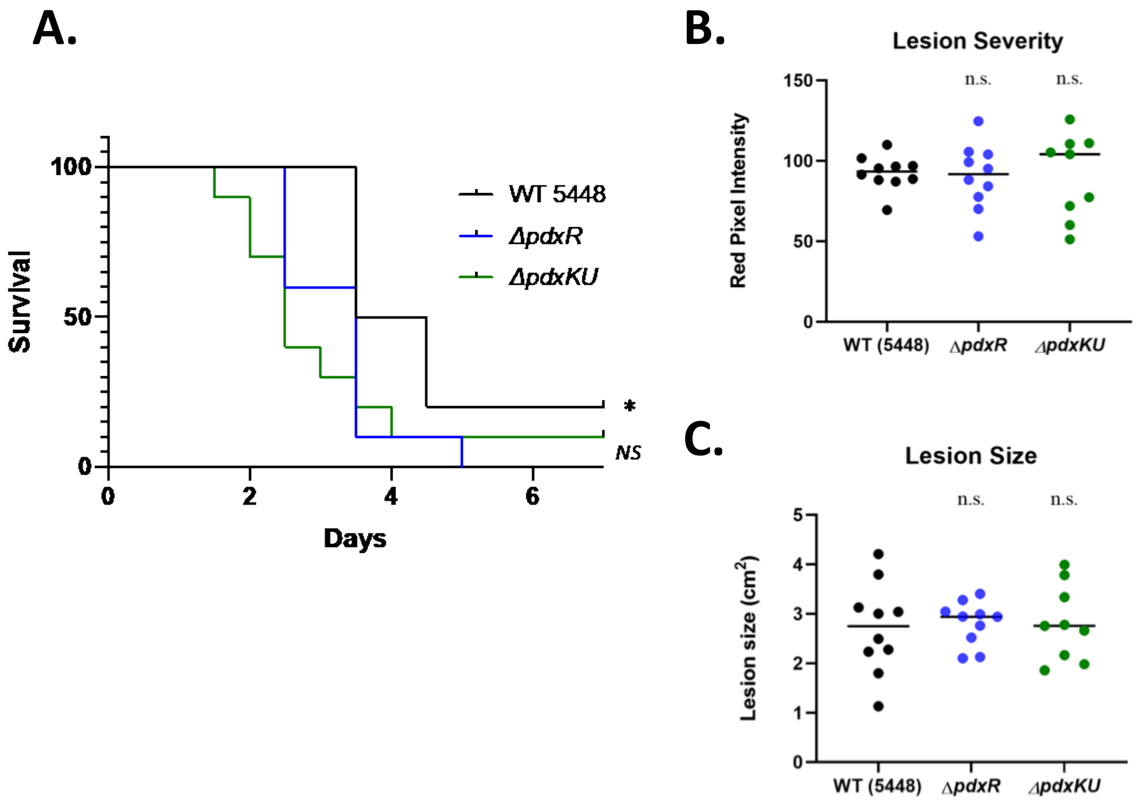

Supplemental Figure S7. Δ*pdxKU* has a modest increase in virulence in comparison to WT in a mouse model of soft tissue infection. WT (5448, black) or the ΔpdxR (blue) and ΔpdxKU (green) mutant GAS were injected subcutaneously (2 x 10^7 CFU) into CD-1 outbred mice. **(A)** Mice were monitored for morbidity over the course 7 days. Lesions were imaged 48 hours post-infection and **(B)** severity (red pixel intensity) and **(C)** size were quantified. Significance was measured via Kaplan-Meier survival analysis **(A)** and the log rank test **(B,C)** (*: P value < 0.05, NS: non-significant).
